# A Water-Promoted Mars−van Krevelen Reaction Dominates Low-Temperature CO Oxidation over Au-Fe_2_O_3_ but Not over Au-TiO_2_

**DOI:** 10.1021/acscatal.3c05978

**Published:** 2024-02-14

**Authors:** Alexander Holm, Bernadette Davies, Sara Boscolo Bibi, Felix Moncada, Joakim Halldin-Stenlid, Laurynas Paškevičius, Vincent Claman, Adam Slabon, Cheuk-Wai Tai, Egon Campos dos-Santos, Sergey Koroidov

**Affiliations:** † Department of Physics, AlbaNova University Center, Stockholm University, 106 91 Stockholm, Sweden; ‡ Department of Materials and Environmental Chemistry, Stockholm University, Svante Arrhenius väg 16C, 114 18 Stockholm, Sweden; § Laboratory of Organic Electronics, Department of Science and Technology (ITN), Linköping University, Norrköping, SE-60174 Sweden; ∥ KBR, Inc., Intelligent Systems Division, NASA Ames Research Center, Moffett Field, California 94035, United States; ⊥ Inorganic Chemistry, University of Wuppertal, Gaußstr. 20, 42119 Wuppertal, Germany; # Advanced Institute for Materials Research (WPI-AIMR), Tohoku University, Aoba-ku, Sendai 980-8577, Japan

**Keywords:** CO oxidation, Mars−van Krevelen, Langmuir−Hinshelwood, mechanism, Au, Fe_2_O_3_, TiO_2_

## Abstract

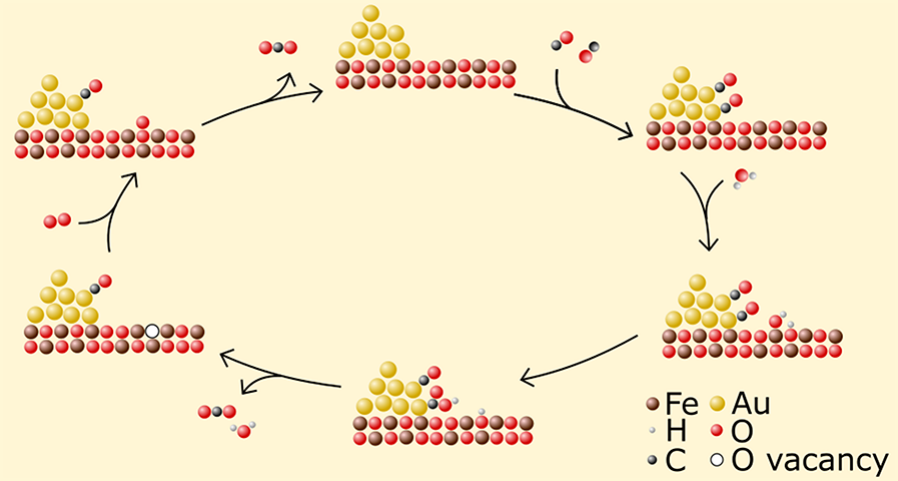

We provide experimental evidence that is inconsistent with often proposed Langmuir−Hinshelwood (LH) mechanistic hypotheses for water-promoted CO oxidation over Au–Fe_2_O_3_. Passing CO and H_2_O, but no O_2_, over Au-γ-Fe_2_O_3_ at 25 °C, we observe significant CO_2_ production, inconsistent with LH mechanistic hypotheses. Experiments with H_2_^18^O further show that previous LH mechanistic proposals cannot account for water-promoted CO oxidation over Au-γ-Fe_2_O_3_. Guided by density functional theory, we instead postulate a water-promoted Mars–van Krevelen (w-MvK) reaction. Our proposed w-MvK mechanism is consistent both with observed CO_2_ production in the absence of O_2_ and with CO oxidation in the presence of H_2_^18^O and ^16^O_2_. In contrast, for Au-TiO_2_, our data is consistent with previous LH mechanistic hypotheses.

CO oxidation over Au nanoparticles (NPs) on metal oxides is intensely studied in heterogeneous catalysis.^1−21^ Water strongly promotes this reaction, making catalysts up to one order of magnitude more active at room-temperature.^9,11−14,16,18,22^ However, there is still much debate regarding the mechanism behind water-promotion. At the center of this debate is whether support lattice-oxygen takes an active part in the reaction (a Mars–van Krevelen mechanism, MvK) or if the reaction takes place exclusively between coadsorbed reaction intermediates (a Langmuir–Hinshelwood mechanism, LH).

With support from theory,^1,20,23^ spectroscopy,^2,4,5,7,24^ temporal analysis of products,^8,9^ and isotope exchange experiments,^4,25^ it has been argued that room-temperature CO oxidation occurs via a lattice-mediated MvK reaction over Au-TiO_2_ and Au-Fe_2_O_3_. However, as pointed out by Chandler et al.,^11^ current mechanistic proposals involving the support lattice do not clearly account for water-promotion. In contrast, various LH-mechanisms have been proposed that could account for this promotion.^11−17^ In particular, two influential studies have proposed that an LH-type mechanism can rationalize water-promotion on both nonreducible (e.g., Al_2_O_3_) and reducible (e.g., TiO_2_, Fe_2_O_3_) supports.^11,12^

Chandler et al. observed that, at room-temperature, CO and O_2_ rate orders, kinetic isotope (hydrogen/deuterium) effects, and H_2_O adsorption were similar over Au-Al_2_O_3_ and Au-TiO_2_, implying that the catalysts run by the same mechanism.^11^ It was argued that because lattice-oxygen on nonreducible Al_2_O_3_ cannot directly participate in the reaction mechanism, and because the data implied that Au-Al_2_O_3_ and Au-TiO_2_ operate by the same mechanism, lattice-oxygen does not participate in room-temperature water-promoted CO oxidation over Au-TiO_2_. Chandler therefore proposed that water-promotion over Au-TiO_2_ and Au-Al_2_O_3_ occurs by an LH-mechanism, with CO adsorbing on Au NPs, and H_2_O adsorbing on the Au-support interface, there promoting O_2_ activation to form a reactive hydroperoxy species (OOH) which then reacts with adsorbed CO to form CO_2_ (Scheme 1).^11^ In this reaction scheme, no oxygen from water would be incorporated into CO_2_, as the water-promotion occurs through a series of proton transfers only. Recently, the LH-mechanism proposed by Chandler for Au-TiO_2_ and Au-Al_2_O_3_ was also proposed to account for water-promotion over Au-Fe_2_O_3_.^14^

**Scheme 1:**
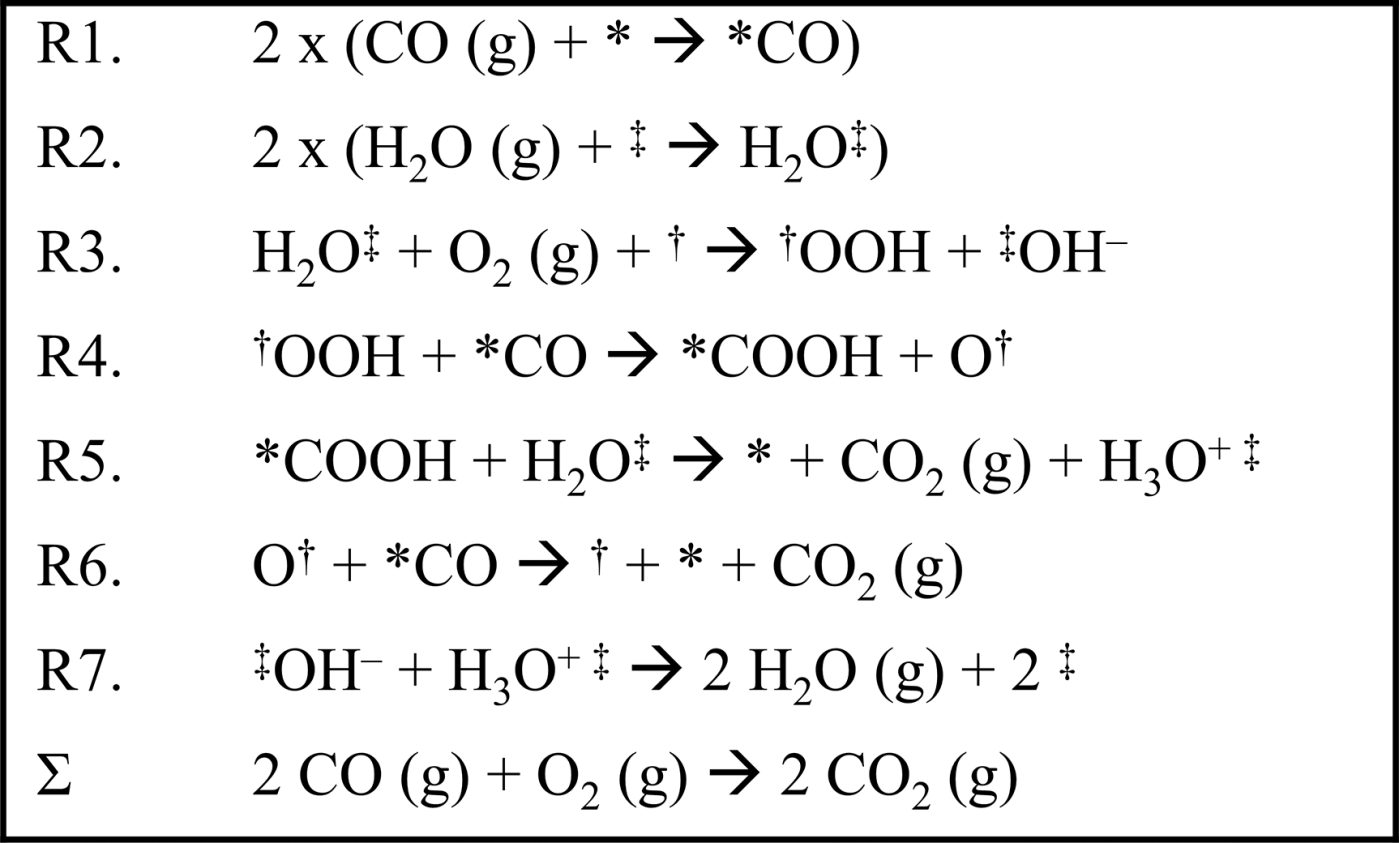
Elementary Reaction Steps for the Postulated (by Chandler et al.)^11^ LH Reaction Mechanism of Water-Promoted CO Oxidation over Au-Al_2_O_3_ and Au-TiO_2_, Which Has Also Been Proposed^14^ to Be Dominant on Au-Fe_2_O_3_ Note: * denotes an active site on the Au NP, away from the NP–support interface. ^†^ denotes a Au site at the NP–support interface, and ^‡^ denotes a support site at the NP–support interface. Formal charges that form during the reaction are assumed to be balanced by formal charges distributed over the Au NP.^11^

Before Chandler, Iglesia et al. postulated a related LH-mechanism for water-promoted CO oxidation over Au-Al_2_O_3_, Au-TiO_2_, and Au-Fe_2_O_3_ (Scheme 2).^12^ Similar to Chandler, it was hypothesized that only adsorbed intermediates participate in the reaction, and that water-promotion occurs because water activates O_2_ to form OOH (Scheme 2). Observing that the rate equations derived from this mechanism accurately predicted rate orders and rates over all catalysts (Au-Al_2_O_3_, Au-TiO_2_, and Au-Fe_2_O_3_), the authors proposed that this LH-mechanism was dominant over the catalysts.^12^

**Scheme 2:**
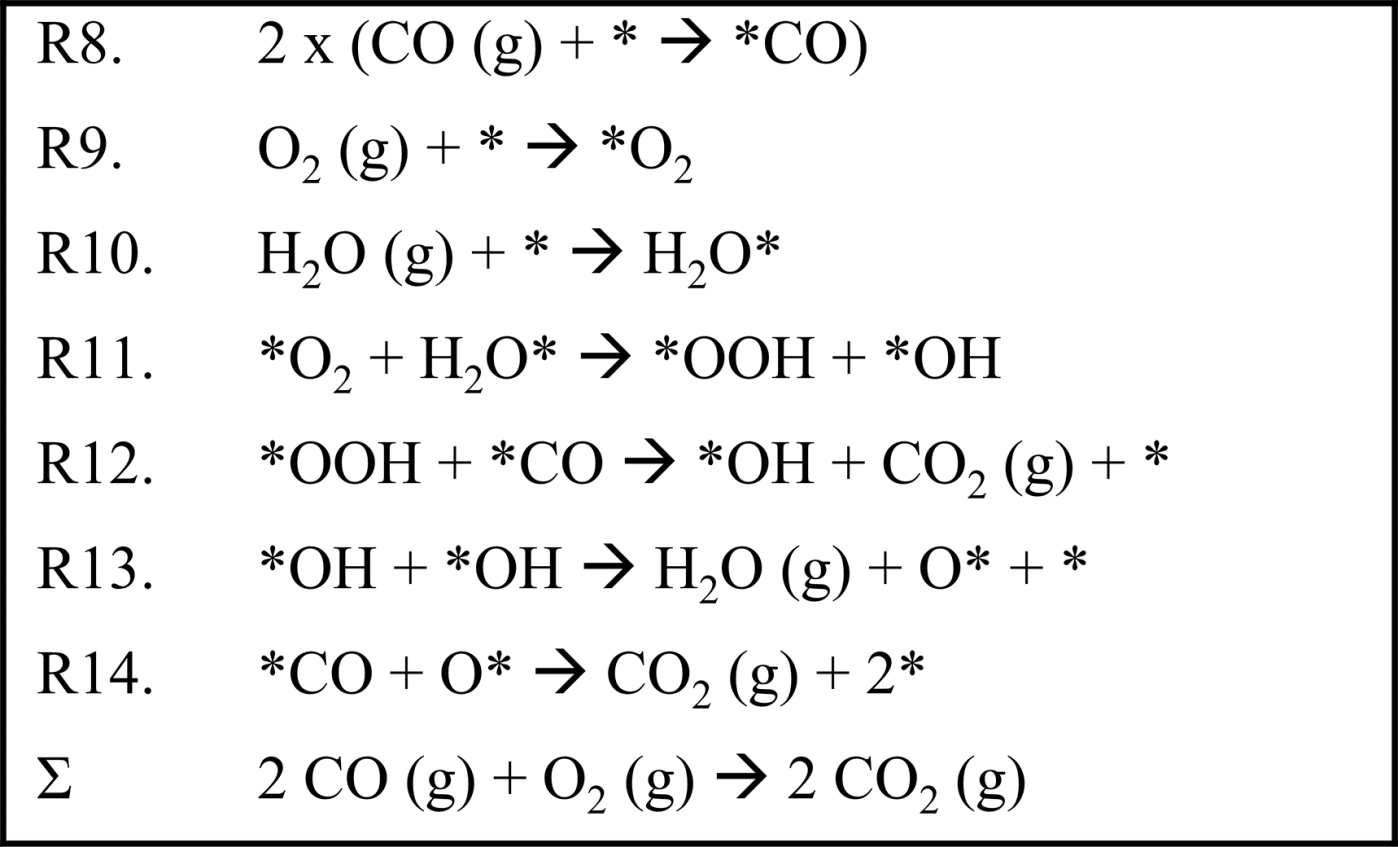
Elementary Reaction Steps for a Previously Postulated (by Iglesia et al.)^12^ LH Reaction Mechanism of Water-Promoted CO Oxidation over Au-Al_2_O_3_, Au-TiO_2_, and Au-Fe_2_O_3_ Note: * denotes an active site on the Au NP.

Here we provide experimental data clearly demonstrating that an LH-type mechanism cannot dominate water-promoted CO oxidation over Au-γ-Fe_2_O_3_ at room-temperature. Instead, we postulate that a lattice-mediated mechanism, which does account for water-promotion, dominates. We present density functional theory (DFT) calculations and careful CO oxidation experiments with C^16^O, ^16^O_2_, and H_2_^18^O suggesting that this mechanism can rationalize water-promoted CO oxidation over Au-γ-Fe_2_O_3_.

We prepared Au-TiO_2_ and Au-γ-Fe_2_O_3_ catalysts (Figure 1A–D) by well-established protocols (Supporting Information, Section S1), resulting in Au NP sizes of 4.3 nm (Au-TiO_2_) and 4.8 nm (Au-γ-Fe_2_O_3_), similar to previous reports.^12,21,26−29^ The Au-γ-Fe_2_O_3_ catalyst was characterized in more detail and consists of polyhedral Au NPs supported on γ-Fe_2_O_3_, where the Au(111) facet is dominating at the Au/γ-Fe_2_O_3_ perimeter, Figure 1E–G. Investigation by high-resolution STEM (Figure S11) found no evidence of single Au atoms, suggesting that Au NPs are the dominant Au species in the catalyst.

**Figure 1:**
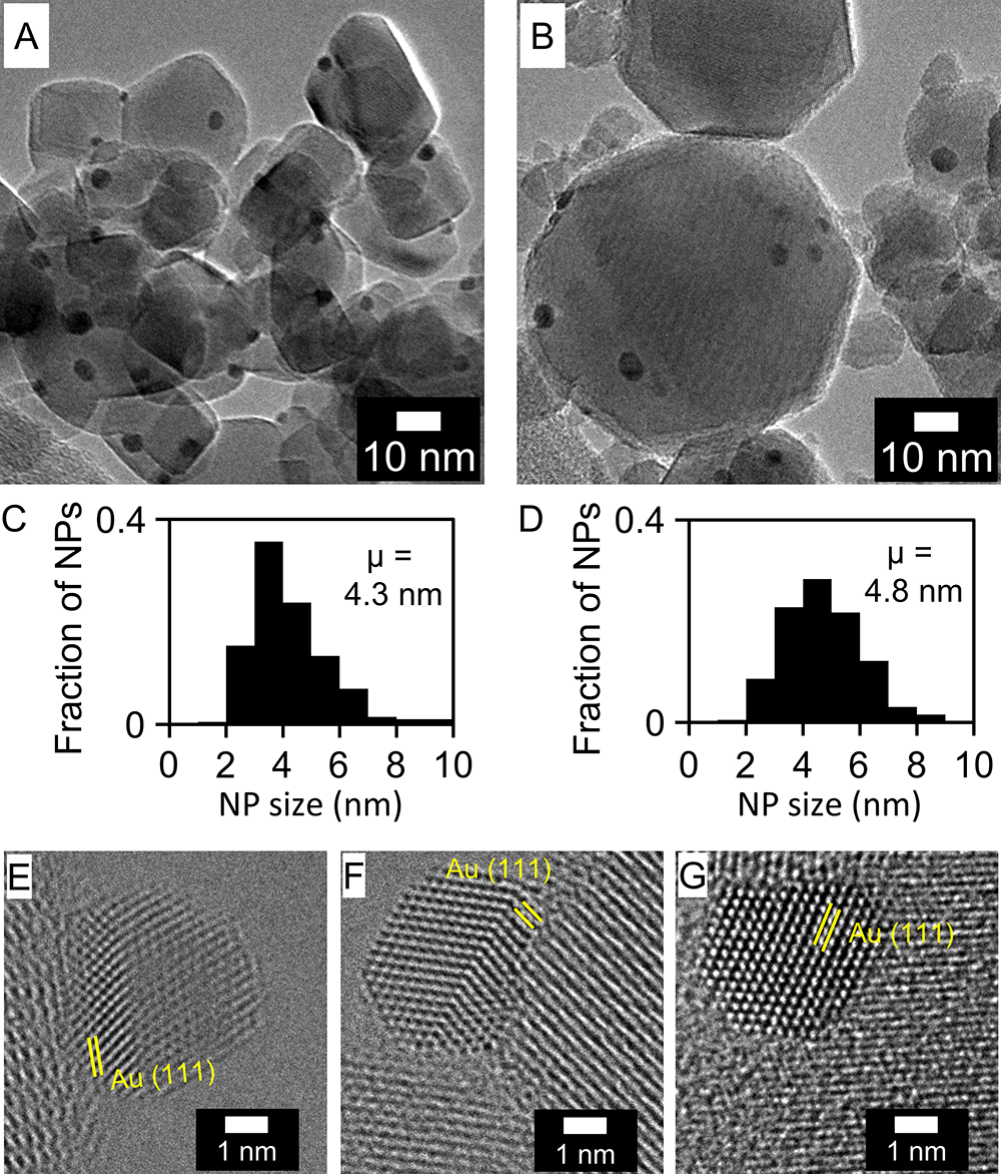
TEM micrographs of (A) Au-TiO_2_ and (B) Au-γ-Fe_2_O_3_. Au NP size histograms for (C) Au-TiO_2_ and (D) Au-γ-Fe_2_O_3_. (E–G) Typical high-resolution TEM images of Au-γ-Fe_2_O_3_. The Au NPs are polyhedral, with the (111) facet dominating at the Au/γ-Fe_2_O_3_ interface.

Catalytic tests were run at ambient temperature (controlled at 25 °C), consistent with previous studies.^2,4,5,11−14,21,27,28,30−33^ Ambient oxygen adsorbed to the catalyst was removed by passing humidified N_2_ (2.8 vol % H_2_O) over the catalyst for 16 h. We then introduced CO (1 vol %) in the inlet flow. Over Au-TiO_2_, no significant CO_2_ production was observed (Figure 2A), while over Au-γ-Fe_2_O_3_, CO_2_ production quickly increased to 0.95 mmol CO_2_ (mol Au)^−1^ s^–1^, then rapidly decreased (Figure 2B). The background O_2_ concentration in our reactor was ∼4 ppm, which was about 2% of the CO_2_ concentration resulting from CO oxidation (Figures S6 and S7). In addition, no H_2_ was produced (Figure S8), ruling out the possibility that the water gas shift reaction (CO + H_2_O → CO_2_ + H_2_) contributes to CO_2_ production. Thus, over Au-γ-Fe_2_O_3_, water-promoted CO oxidation in the absence of O_2_ occurs with removal of oxygen from the γ-Fe_2_O_3_ lattice. Of course, the CO_2_ production in the absence of O_2_ is not stable because the γ-Fe_2_O_3_ lattice is gradually depleted of oxygen.

**Figure 2:**
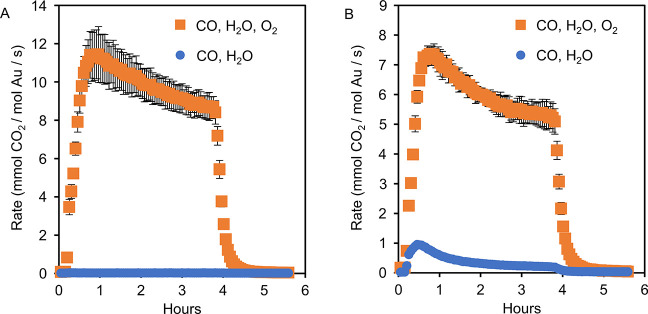
(A) Transient CO oxidation rates over Au-TiO_2_. (B) Transient CO oxidation rates over Au-γ-Fe_2_O_3_. Reaction starts immediately upon introduction of CO and terminates immediately upon removal of CO (at 3 h 45 min). Orange squares: 1 vol % CO, 2.8 vol % H_2_O, 20 vol % O_2_, balance N_2_. Blue circles: 1 vol % CO, 2.8 vol % H_2_O, balance N_2_. Reaction temperature was 25 °C and pressure 1 atm. The gas hourly space velocity (GHSV) was 21 L h^–1^ g^–1^_cat_, and the CO conversion was below 20% (ensuring data was collected under differential conditions).^13,37^ Reported curves are averages of three independent measurements. Error bars are 2 standard deviations wide. For some data points, the error bars are so small, they are obscured by the data labels. Full data sets in Figure S9.

Thus, water-promoted CO oxidation over Au-γ-Fe_2_O_3_ can proceed by abstraction of lattice-oxygen, while over Au-TiO_2_, this appears unlikely. Our data, therefore, does not refute previously proposed LH-mechanisms over Au-TiO_2_. However, over Au-γ-Fe_2_O_3_, we need to investigate how significant the lattice reaction is when O_2_ is present. To probe this question, we assessed water-promoted CO oxidation with O_2_, for both Au-TiO_2_ (Figure 2A) and Au-Fe_2_O_3_ (Figure 2B). The rates measured are similar to previously reported rates,^2,4,5,21,27,28,30−33^ showing that our catalysts are comparable to previously reported catalysts. Over Au-γ-Fe_2_O_3_, the maximum rate in the presence of water, but without O_2_ (0.95 mmol CO_2_ (mol Au)^−1^ s^–1^), is ≈13% of the maximum rate with O_2_. This suggests that a lower bound of ≈13% CO_2_ is produced by a water-promoted lattice-route during CO oxidation over Au-γ-Fe_2_O_3_. However, the true contribution is likely higher, since with O_2_ present, lattice-oxygen is continuously replenished. Below, we further investigate what this true contribution may be, using a combination of DFT calculations and CO oxidation experiments in the presence of H_2_^18^O.

To rationalize the observed CO_2_ production over Au-γ-Fe_2_O_3_ in the absence of O_2_, a lattice reaction (MvK) mechanism must be invoked. Considering the exposed Au(111) facets (Figure 1E–G), a suitable Au-γ-Fe_2_O_3_ model system was created (Supporting Information, Section S2), using DFT. Using this system, we found a low-barrier, water-promoted MvK (w-MvK) mechanism, presented in Scheme 3 and Figure 3. A similar mechanism has been proposed for water-promoted CO oxidation over single-atom Pt_1_-CeO_2_,^34^ and we propose that this mechanism can be adapted to rationalize room-temperature CO oxidation over Au-γ-Fe_2_O_3_.

**Scheme 3:**
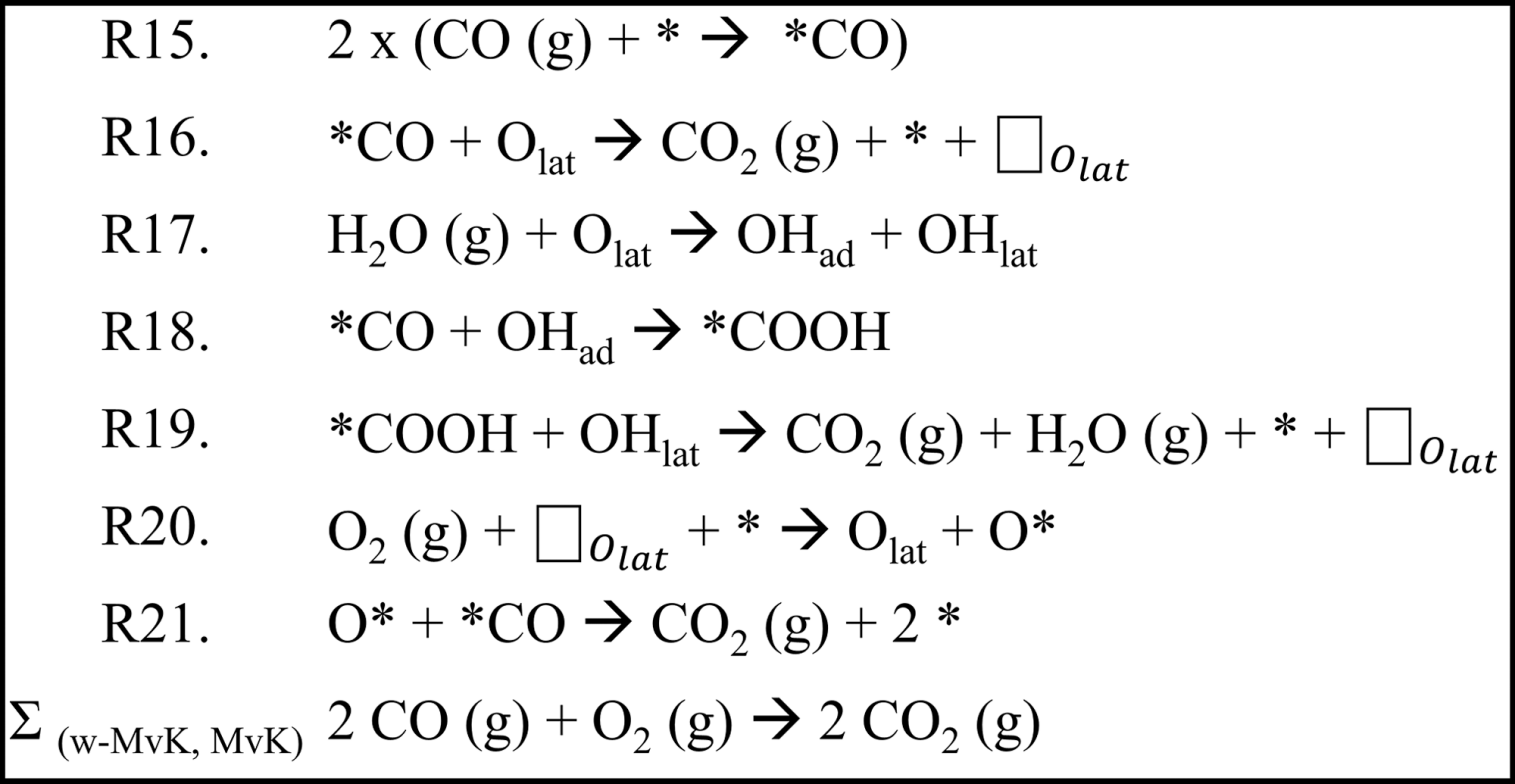
Elementary Reaction Steps for Our Proposed w-MvK CO Oxidation Mechanism (R15, R17–R21); the (Non-Feasible) Non-Water-Promoted MvK Mechanism Is Represented by R15, R16, R20, and R21 Note: * denotes an active site on the Au NP. O_lat_ denotes a lattice-oxygen near the Au NP. □_*O*_lat__ denotes a lattice-oxygen vacancy. OH_ad_ denotes a hydroxyl on a lattice Fe, and OH_lat_ denotes a hydroxyl in a lattice-oxygen position.

**Figure 3:**
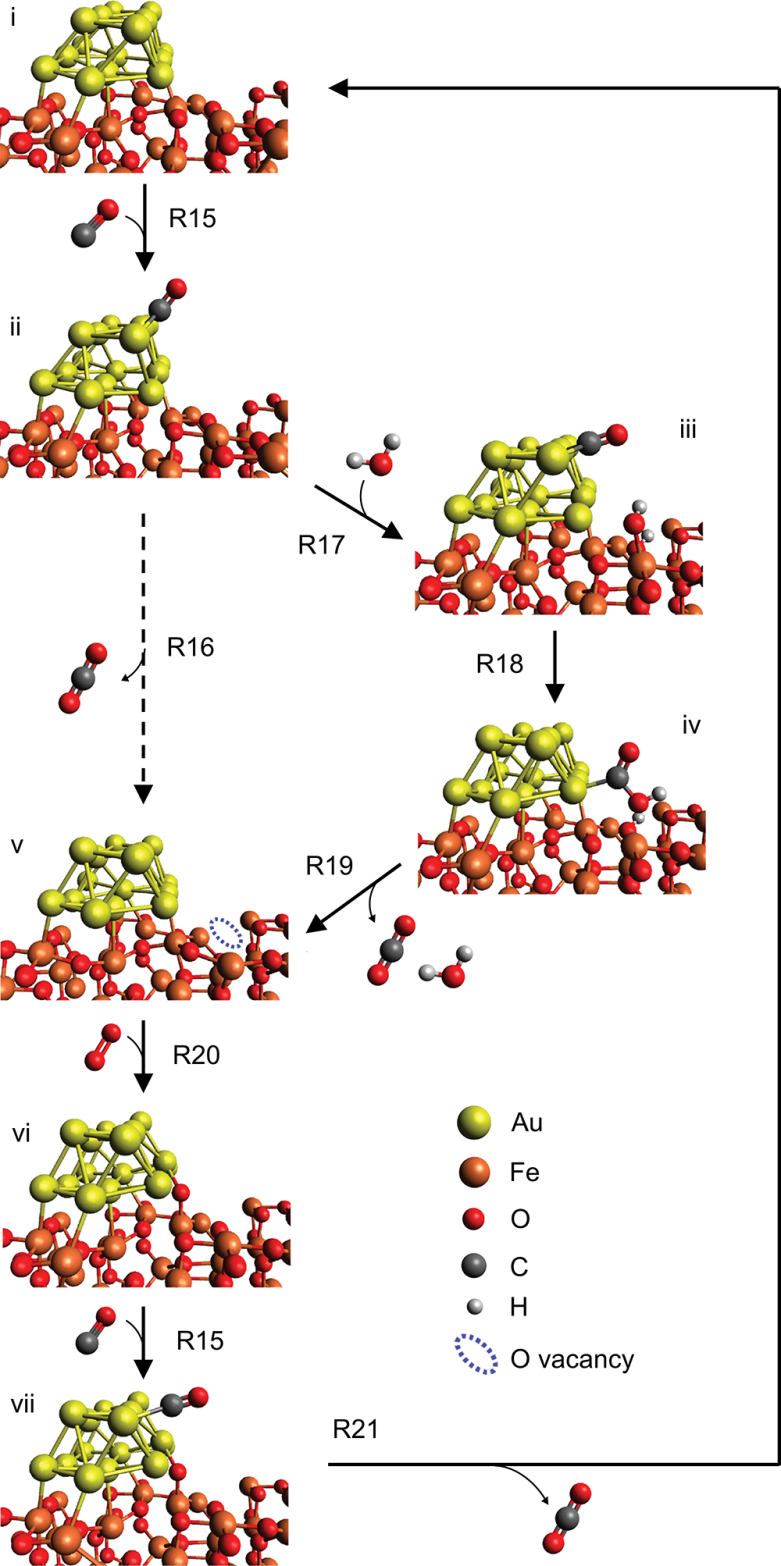
Schematic representation of our proposed mechanism for w-MvK CO oxidation (R15, R17–R21) over Au-γ-Fe_2_O_3_. The non-feasible, non-water-promoted, MvK mechanism is represented by R15, R16, R20, and R21.

Our DFT calculations suggest that direct abstraction of γ-Fe_2_O_3_ lattice-oxygen by Au-adsorbed CO comes with a prohibitively high barrier of 0.98 eV (reaction R16). However, introduction of water presents a low-barrier alternative (to R16), but likewise lattice-mediated, path for CO oxidation (R17–R19, Scheme 3). Reaction R17 describes dissociative adsorption of H_2_O onto an Fe_lat_–O_lat_ motif in the γ-Fe_2_O_3_ surface, with H_2_O splitting into a hydroxyl on top of Fe_lat_ (OH_ad_) and a lattice-hydroxyl (OH_lat_) (Scheme 3 and Figure 3). Reaction R18 describes the reaction of CO, adsorbed on gold, with the water-derived OH_ad_ to form a carboxylic group (COOH) adsorbed to the Au NP. This carboxylic group then reacts (R19) with the OH_lat_ formed during water dissociation (R17) to produce CO_2_ and re-form H_2_O, thereby forming a lattice-oxygen vacancy. Note from reactions R17–R19 that water is first consumed, and then regenerated, such that no net-consumption of water occurs, and water acts purely as a promoter. Also note that, although oxygen from water is incorporated into CO_2_, water is regenerated with oxygen from the γ-Fe_2_O_3_ lattice, meaning that overall, reactions R17–R19 yield the same stoichiometry as reaction R16 (Scheme 3 and Figure 3). The critical point is that the water-mediated abstraction of lattice-oxygen (in the form of OH_lat_) is energetically more favorable than O_lat_ abstraction in the absence of water. Between reactions R17–R19, reaction R17 has the highest barrier (0.44 eV), to compare with the barrier for direct abstraction of O_lat_ by CO (R16, 0.98 eV). Reaction R20 describes the filling of an oxygen vacancy with oxygen from O_2_ (g), forming an oxygen atom (O*) on the Au NP in the process. The catalytic cycle closes (R21) with Au-adsorbed CO reacting with this oxygen atom to form CO_2_. These final steps (R20, R21) of the catalytic cycle are essentially without barriers (Figure 4).

**Figure 4:**
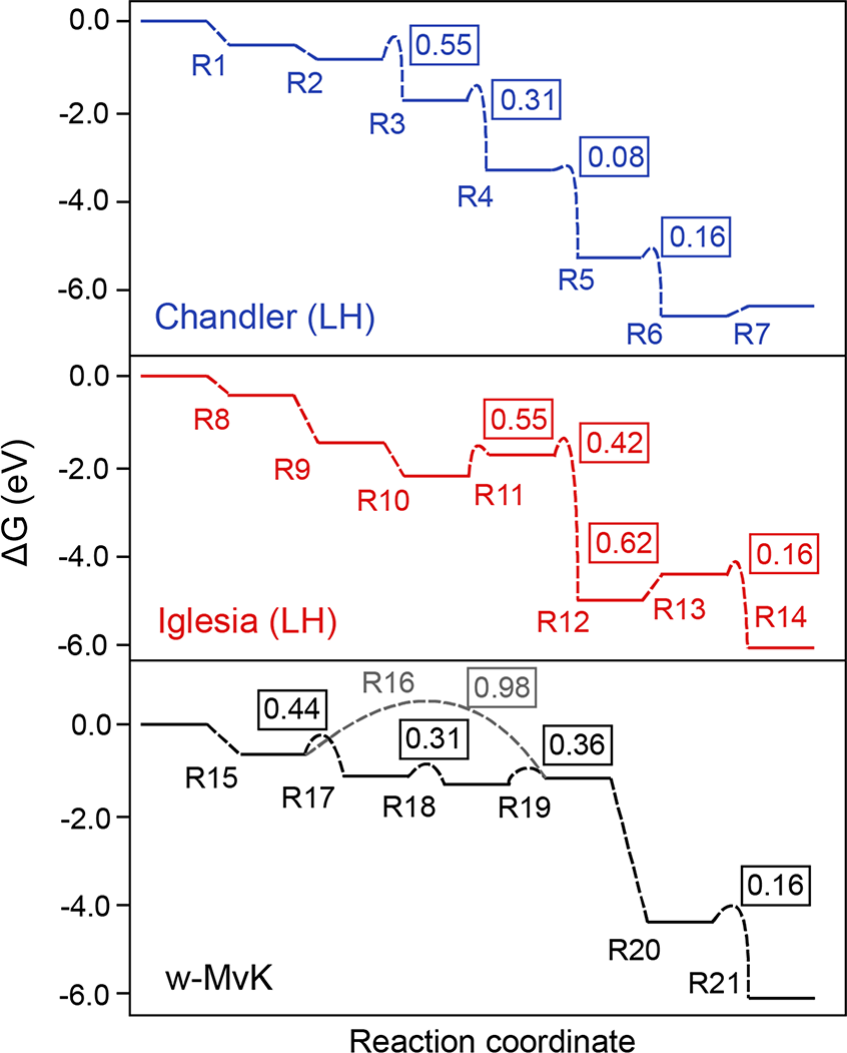
Energy diagrams of previously proposed LH-mechanisms and of our proposed w-MvK mechanism. The reactions are labeled according to Schemes 1–3. Boxed values (in eV) are activation energies for the respective elementary reaction. Refer to the Supporting Information, Section S2, for computational details.

To compare our proposed w-MvK mechanism (Scheme 3) with previously proposed LH-mechanisms (Schemes 1 and 2), we also calculated reaction barriers for these LH-mechanisms (Figure 4). In Chandler’s proposed mechanism, we find that the rate-determining step is O_2_ (g) activation by water to form OOH (R3, Scheme 1), with a barrier of 0.55 eV. This same step is also rate-determining in Iglesia’s proposed mechanism (R11, Scheme 2). However, in this mechanism, another step (R13, Scheme 2) is also rate-limiting (reaction barrier of 0.62 eV), namely, the recombination of two adsorbed hydroxyl-groups to form water and adsorbed atomic oxygen. Our calculations therefore indicate that the w-MvK mechanism may be favored over these previously proposed LH-mechanisms. However, the similar reaction barriers suggest that an LH-mechanism and an MvK mechanism potentially could operate simultaneously over Au-γ-Fe_2_O_3_ (although, as shown above, a lower bound of 13% CO_2_ must result from an MvK mechanism). To further distinguish between the mechanisms, we turned to experiments of H_2_^18^O-promoted CO oxidation.

Different mechanisms of H_2_^18^O-promoted C^16^O oxidation would lead to different abundances of C^16^O_2_, C^16^O^18^O, and C^18^O_2_. Chandler’s mechanism would lead to no ^18^O incorporation into CO_2_ (100% C^16^O_2_ expected). In contrast, Iglesia’s mechanism would lead to 75% C^16^O_2_ and 25% C^16^O^18^O, while our proposed w-MvK mechanism should result in relative abundances of 50% C^16^O_2_ and 50% C^16^O^18^O. Please refer to the Supporting Information, Section S4, for a detailed analysis of how these relative isotopic CO_2_ abundances can be predicted from the respective mechanisms.

Before we discuss the experimentally observed relative abundances, we note that CO_2_ also exchanges oxygen directly with H_2_^18^O.^35^ This exchange occurs in the reactor piping and on the catalyst bed and is therefore a reactor-specific property that must be deconvoluted from the abundances resulting from ^18^O insertion into CO_2_ due to the CO oxidation mechanism. To estimate the ^18^O exchange between H_2_^18^O and different isotopic CO_2_ species in our reactor, we prepared a mixture of C^16^O_2_, H_2_^18^O, and ^16^O_2_ with the same concentrations as obtained during CO oxidation. We then passed this mixture over the Au-γ-Fe_2_O_3_ catalyst, at the same conditions used for C^16^O oxidation. This experiment allowed us to deduce (Supporting Information, Section S4) the reactor-specific probabilities that a given CO_2_ species (C^16^O_2_, C^16^O^18^O, or C^18^O_2_) transforms to another isotopic CO_2_ species by ^18^O exchange with H_2_^18^O:
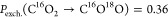
1
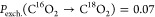
2
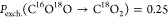
3With knowledge of our reactor-specific ^18^O incorporation into different CO_2_ species by exchange with H_2_^18^O, and with the abundances predicted to result from the different CO oxidation mechanisms, we can now predict, for each hypothetical mechanism, the expected abundances in the reactor effluent. For Iglesia’s LH-mechanism (Scheme 2), we would expect 43% C^16^O_2_, 46% C^16^O^18^O, and 11% C^18^O_2_. For Chandler’s LH-mechanism (Scheme 1), we would expect 58% C^16^O_2_, 36% C^16^O^18^O, and 7% C^18^O_2_. Finally, for our proposed w-MvK mechanism, we would expect 29% C^16^O_2_, 56% C^16^O^18^O, and 16% C^18^O_2_. Please refer to the Supporting Information, Section S4, for a detailed calculation of these predicted abundances in the reactor effluent.

Transient CO oxidation experiments over Au-γ-Fe_2_O_3_, using C^16^O, ^16^O_2_, and H_2_^18^O, were then carried out (Figure 5) using the conditions described in Figure 2 (although GHSV was reduced, through the flow-rate, to allow prolonged bubbling through a small volume of H_2_^18^O). During CO oxidation, we measured transient production rates (Figure 5A) of C^16^O_2_, C^16^O^18^O, and C^18^O_2_ and then calculated their transient relative abundances in the reactor effluent (Figure 5B). The experimental abundances thus found were 28% C^16^O_2_, 55% C^16^O^18^O, and 17% C^18^O_2_ (Figure 5 C). It is noteworthy that, although the catalyst deactivates during the reaction, the relative isotopic abundances remain constant, suggesting that the same mechanism is dominant during the entire course of reaction.

**Figure 5:**
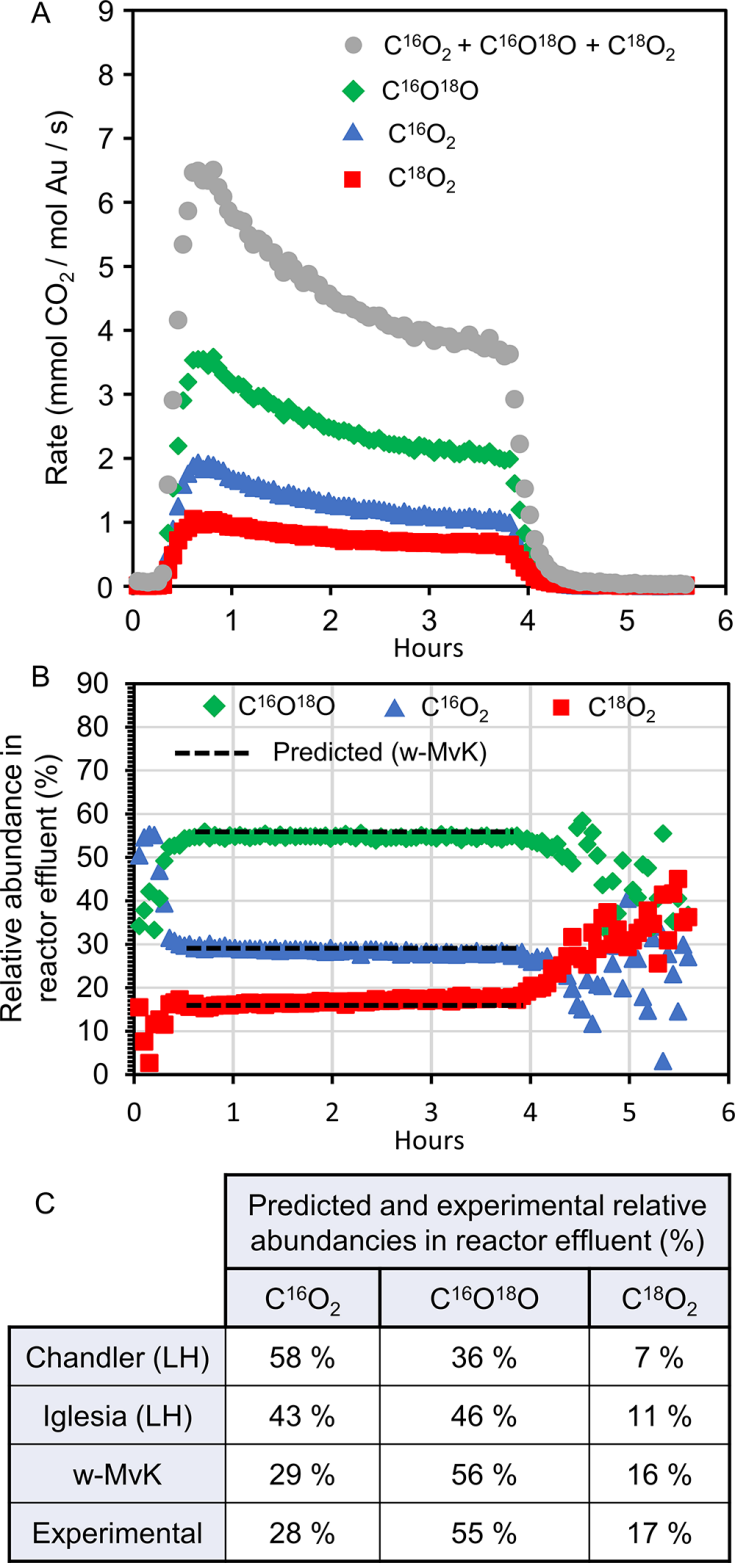
C^16^O oxidation over Au-γ-Fe_2_O_3_ with H_2_^18^O and ^16^O_2_. (A) Transient production rates of different isotopic CO_2_ species, and the total rate. (B) Transient relative abundances of different isotopic CO_2_ species in the reactor effluent. Relative abundances were calculated (e.g., for C^16^O_2_) as. The relative abundances predicted from our proposed w-MvK mechanism are indicated by dashed lines. (C) Comparison between predicted (from different mechanistic hypotheses) and experimentally observed relative abundances in the reactor effluent. Reaction conditions: 1 vol % C^16^O, 2.8 vol % H_2_^18^O, 20 vol % ^16^O_2_, balance N_2_. Reaction temperature was 25 °C, pressure 1 atm, and space velocity (GHSV) 10.5 L h^–1^ g^–1^_cat_.

By comparing (Figure 5C) the measured isotopic CO_2_ abundances during reaction with the abundances predicted by the previously proposed LH-mechanisms (Schemes 1 and 2), it is clear that these mechanisms are not consistent with the observed abundances in the reactor effluent. In contrast, our proposed w-MvK mechanism predicts the observed isotopic CO_2_ abundances to within one percentage point (Figure 5C). Put differently, the observed isotopic CO_2_ abundances—after correcting for ^18^O exchange between CO_2_ and H_2_^18^O—suggest that H_2_^18^O-promoted CO oxidation over Au-γ-Fe_2_O_3_ results in 50% C^16^O_2_ and 50% C^16^O^18^O. This is clearly inconsistent with previous LH-mechanisms^11,12^ but is consistent with our proposed w-MvK mechanism.

In conclusion, since no significant lattice reaction is observed during water-promoted CO oxidation over Au-TiO_2_, previously proposed LH-mechanisms^11−13,16,17^ remain plausible for this system, at least at room-temperature. However, for room-temperature, water-promoted CO oxidation over Au-γ-Fe_2_O_3_, there is a lattice reaction that significantly contributes to the activity (Figure 2B). By accounting for ^18^O exchange between CO_2_ and H_2_^18^O in H_2_^18^O-promoted CO oxidation, we find that the reaction mechanism leads to isotopic abundances of 50% C^16^O_2_ and 50% C^16^O^18^O. For Au-γ-Fe_2_O_3_, these results specifically challenge two previously proposed LH-mechanisms^11,12^ (Schemes 1 and 2) and also other^14,19,36^ previous LH-mechanistic proposals. In contrast, our proposed w-MvK mechanism (Scheme 3 and Figure 3) is consistent with the data and constitutes a useful mechanistic hypothesis.

We have shown that our w-MvK mechanism can rationalize room-temperature, water-promoted CO oxidation over Au-γ-Fe_2_O_3_. However, we also believe this mechanism could rationalize CO oxidation in nominally dry conditions. In catalytic tests at room-temperature, nominally dry gas streams will contain 1–10 ppm H_2_O,^37^ which—because water is not consumed—likely is sufficient for water-promotion.^12,16,22,37^ Furthermore, our DFT calculations indicate that CO abstraction of lattice-oxygen in absence of water is implausible (R16, 0.98 eV) compared to the water-promoted abstraction (R15, R17–R21) with a rate-determining barrier of 0.44 eV. We therefore hypothesize that our w-MvK mechanism could rationalize observations^2,3,5^ of a lattice reaction in nominally water-free CO oxidation over Au-γ-Fe_2_O_3_. In addition, because many studies show that a lattice reaction occurs also on Au-α-Fe_2_O_3_,^2−5,9,26,38^ we hypothesize that our proposed mechanism could operate also in these systems. We therefore hypothesize that our proposed w-MvK mechanism could rationalize CO oxidation over many reported Au-Fe_2_O_3_ catalysts both in nominally dry and in humid conditions.

Finally, we note that although our data does not support a w-MvK mechanism over Au-TiO_2_ at room-temperature, there is convincing literature evidence that an MvK-type mechanism occurs at slightly elevated temperatures.^23,39−42^ It may therefore be interesting to evaluate our proposed w-MvK mechanism also for high-temperature water-promoted CO oxidation over Au-TiO_2_.

## Supporting Information Available

Section S1: experimental details; Section S2: computational details and results; Section S3: additional figures; and Section S4: calculations of the predicted abundances of C^16^O_2_, C^16^O^18^O, and C^18^O_2_ resulting from C^16^O oxidation with ^16^O_2_ and H_2_^18^O over Au-γ-Fe_2_O_3_ (PDF)
