# Supplementary material for: A Water-Promoted Mars−van Krevelen Reaction Dominates Low-Temperature CO Oxidation over Au-Fe2O3 but Not over Au-TiO2
Source: ACS Catal. 2024 Feb 14;14(5):3191–7. doi: 10.1021/acscatal.3c05978 (PMC10913026; doi:10.1021/acscatal.3c05978)
Supplement: Supplementary file 1 — cs3c05978_si_001.pdf [file cs3c05978_si_001.pdf]

Supporting Information for

A water-promoted Mars-van-Krevelen reaction dominates low-temperature CO oxidation over Au-Fe_2O_3, but not over Au-TiO_2

Alexander Holm[a,b,c],‡ Bernadette Davies[a,b],‡ Sara Boscolo Bibi[a], Felix Moncada[a], Joakim Halldin-Stenlid[d], Laurynas Paškevičius[a], Vincent Claman[a], Adam Slabon[a,e], Cheuk-Wai Tai[b], Egon Campos dos-Santos[f], Sergey Koroidov[a,*]

[a]Department of Physics, AlbaNova University Center, Stockholm University, 10691 Stockholm, Sweden
[b]Department of Materials and Environmental Chemistry, Stockholm University, Svante Arrhenius väg 16C, 114 18 Stockholm, Sweden
[c] Laboratory of Organic Electronics, Department of Science and Technology (ITN), Linköping University, Norrköping, SE-60174 Sweden
[d]KBR, Inc., Intelligent Systems Division, NASA Ames Research Center, Moffett Field, California 94035, United States
[e]Inorganic Chemistry, University of Wuppertal, Gaußstr. 20, 42119 Wuppertal, Germany
[f]Advanced Institute for Materials Research (WPI-AIMR), Tohoku University, Aoba-ku, Sendai 980-8577, Japan

‡ These authors contributed equally to this work

**Corresponding author:*
Sergey Koroidov
Department of Physics, Stockholm University
E-mail: sergey.koroidov@fysik.su.se

Table of contents

S1. Experimental details……………………………………………..………..……….2

S1.1. Chemicals……………………………………………..………..……..2

S1.2. Cleaning procedures……………………………..………..……..2

S1.3. Preparation of catalysts……………………………..……………..2

S1.4. Characterization techniques…………………………….…….………3

S1.5 Catalytic tests………………………………………………….…..……...3

S2. Computational details and results………………………………….………...……5

S2.1. Computational details…………………………………………….…..……5

S2.2. Free-energy calculations……………………..………………….….……...7

S2.3. DFT Reaction energetics…………………...……………………..………..8

S3. Miscellaneous Figures…………………………..……………………..……13

S4. Calculation of predicted abundances of $C^{16}O_2$, $C^{16}O^{18}O$ and $C^{18}O_2$ resulting from $C^{16}O$ oxidation with $^{16}O_2$ and $H_2{}^{18}O$ over Au-γ-Fe_2O_3…………………................……..……19

S1. Experimental details.

S1.1. Chemicals

Water for all synthesis was taken from a Millipore system (MilliQ water). $H_2{}^{18}O$ (97 %), $HAuCl_4$ • 3 H_2O (> 99.9 %), γ-Fe_2O_3 (< 50 nm powder), TiO_2 (21 nm powder, Degussa), urea (99 – 100 %), isopropyl alcohol (> 99.5 %), KOH (90 %) were obtained from Sigma Aldrich. Gas mixtures used: CO (99.7 %, Linde), CO_2 (99.9995 %, Air Products), 1 vol % Kr in N_2 (Air Products), 20 vol % O_2 and 1 vol % Kr in N_2 (Air products).

S1.2. Cleaning procedures

A base bath (8 L of isopropyl alcohol, 2 L of deionized (DI) water, and 500 g of KOH) was used to clean all glassware. After immersion for at least 1 h in the base bath, the glassware was rinsed copiously in DI water and then in Milli-Q water, followed by drying in a clean oven at 80 ^oC.

S1.3.Preparation of catalysts

We adapted a well-established deposition-precipitation protocol from the literature[1] for synthesis of catalysts with 1 % weight loading of Au. Briefly, 0.06 g of $HAuCl_4$ • 3 H_2O was added to a 500 mL reaction flask, using a glass-boat and a Teflon spatula. 300 mL water was then added, and the $HAuCl_4$ • 3 H_2O allowed to dissolve. Urea (8 g) was then added to the reaction flask, and allowed to dissolve, followed by addition of 3 g of the support (γ-Fe_2O_3 or TiO_2) . The solution was then sonicated (Elmasonic P, Elma) for 2 min. The reaction flask was capped with septa, including a 23 gauge needle for venting, and a glass-thermometer inserted through the septa. The solution was then stirred with a magnetic stirbar (700 rpm) and heated to 80 ^oC +/- 3 ^oC in an oil-bath. The reaction was then stirred for 16 hours. The reaction was then brought to 50 ^oC on a water-bath. The suspension was then transferred to falcon tubes and the product washed repeatedly by centrifugation (Heraeus Megafuge 16, Thermo Scientific) followed by re-suspension in water at 50 ^oC:

- Centrifugation at 10000 rpm (3 min), discarding the supernatant followed by re-suspending (sonication) the sample in water at 50 ^oC for 10 min.
- Centrifugation at 10000 rpm (3 min), discarding the supernatant followed by re-suspending (sonication) the sample in water at 50 ^oC for 10 min.
- Centrifugation at 10000 rpm (3 min), discarding the supernatant followed by re-suspending (sonication) the sample in water at 50 ^oC for 10 min.
- Centrifugation at 10000 rpm (3 min), discarding the supernatant followed by re-suspending the sample in water at 50 ^oC for 10 min.
- Centrifugation at 10000 rpm (3 min), collecting the sample and then drying the sample in desiccator for 36 h.

Finally, the sample was annealed in air at 250 °C for 4 hours to produce the catalysts (Au-γ-Fe_2O_3 and Au-TiO_2, respectively, 1 wt % Au.). At all times during sample preparation and storage, the samples were protected from light. BET and XRD analysis of the catalysts are presented in Figure S10.

S1.4. Characterization Techniques

Transmission electron microscopy (TEM) images were collected on a JEOL JEM-2100F instrument operating at 200 kV. The samples were deposited onto lacey carbon / Cu grids by simply shaking the grid in a small amount of sample powder. High-resolution TEM images by aberration-corrected TEM (Thermo Fisher Themis Z) operated at 300 kV. Spherical aberration was compensated using an image corrector (CEOS CETCOR) up to third order. TEM images were acquired by Gatan OneView camera. Nitrogen adsorption isotherms for Brunauer-Emmett-Teller (BET) analysis were recorded on a Micromeritics ASAP2020 analyzer at 77 K. Samples were degassed at 100 °C for 12 hours prior to measurement. Powder X-ray diffraction patterns were recorded on a Bruker D8 DISCOVER diffractometer applying Cu Kα radiation, with a scanning speed of 4° min^{-1} in a 2θ range of 10° – 80° in reflection mode.

S1.5. Catalytic tests

CO oxidation rates were measured in a packed-bed plug flow reactor (quartz tube). The catalyst bed (40 mg catalyst) was placed between 2 layers of acid-washed sand (200 mg bottom layer, 280 mg top layer). Without catalyst, the sand was not active for CO oxidation under the reaction conditions used. All reactions were carried out at ambient pressure, and the reaction temperature was controlled at 25 °C. Gas flows were obtained by mixing CO with either a mixture of 1 vol % Kr in N_2 or with a mixture of 20 vol % O_2 and 1 vol % Kr in N_2, where Kr was used as an inert internal standard. Gases were mixed with electronic mass-flow controllers (EL-FLOW prestige, Bronkhorst). Water was introduced in the gas stream by a bubbler (15 mL H_2O) kept at 23 °C, corresponding to a concentration of 2.8 vol % H_2O. Concentrations in the reactor effluent were measured, in-line, by mass-spectrometry (CIS 300, SRS), using 1 vol % Kr as an internal standard (all m/z signals were normalized to the m/z = 86 signal where the m/z = 86 signal corresponds to ^{86}Kr).

Before measurements, ambient gases adsorbed to the catalyst were removed by passing humidified N_2 (2.8 vol % $H_2{}^{16}O$ or $H_2{}^{18}O$) over the catalyst for 16 h. The reaction mixture was then introduced; {1 vol % CO, 1 vol % Kr, 2.8 vol % H_2O, balance N_2} for measurement in absence of O_2, and {1 vol % CO, 1 vol % Kr, 20 vol % O_2, 2.8 vol % H_2O, balance N_2} for measurement in presence of O_2. The flow rate was 14 mL min^{-1}, corresponding to a gas hourly space velocity (GHSV) of 21 L h^{-1} g^{-1}_{cat}. These conditions led to CO conversions below 20 %, thereby ensuring differential conditions, allowing direct comparison of measured rates.[2,3] For $C^{16}O$ oxidation experiments using $^{16}O_2$ and $H_2{}^{18}O$, the same conditions as described above were used, with the difference that $H_2{}^{16}O$ was replaced by

$H_2{}^{18}O$, and a flow-rate of 7 mL min^{-1} (instead of 14 mL min^{-1}) was used in the $H_2{}^{18}O$ experiments to allow prolonged bubbling through the small volume (1 mL) of $H_2{}^{18}O$, resulting in a GHSV of 10.5 L h^{-1} g$^{-1}_{cat}$.

S2. Computational details and results

S2.1. Computational details

Periodic DFT calculations were performed in Vienna Ab-initio Simulation Package (VASP),[4] employing the PBE[5] exchange-correlation functional. Standard PBE PAW potentials[6] were used to represent core states while the valence states were treated explicit by plane-wave basis-set with an energy cutoff of 520 eV. Γ-centered Monkhorst-Pack type k-points meshes using tetrahedron method and Blöchl corrections.[7] The first Brillouin zone was sampled on (3×3×1) for slab and (1×1×1) for molecules. First-order Methfessel-Paxton[8] scheme with a Gaussian width of 0.15 eV was employed for all structures. Structure relaxations were conducted within the criteria of 10^{-5} eV and 0.015 eV/Å for energy and atom force, respectively. From the optimized bulk structure, γ-Fe_2O_3 and Au surface models were constructed in the atomic simulation environment (ASE).[9] For all slab structures a vacuum space of more than 20 Å was used, and dipole correction was used along slab z-direction.[10] Spin-polarized calculations were carried out, and the most stable spin state for all systems was used in this work. Molecules were optimized in a cubic box of 20 Å. Transition states (TS) were obtained by CI-NEB (Climbing Image - Nudged Elastic Band) method.[11] Nine images were used, and each image converged to the minimum energy pathway using a convergency accuracy of 0.05 eV/Å. The activation energy (E_a) was obtained by the difference between the initial state (IS) and TS total energy, $E_a = E_{TS} - E_{IS}$.

The unit cell used for bulk calculations was obtained by Pecharroman *et al.*[12] crystallographic refinement data. Maghemite (γ-Fe_2O_3) crystalizes at room temperature in a cubic system and belongs to Fd3m space group. The experimental cell parameters were found to be {***a***=***b***=**c**= 8.35 Å, α=β=γ= 90.0°}, while our bulk optimizations found the cell parameters {***a***=***b***=**c**= 8.48 Å, α=β=γ= 90.0°}, within 2% of experimental values. This result clearly shows that DFT/PBE level can describe crystal structure of maghemite.

The lattice parameters of Au (A) and γ-Fe_2O_3 (B) phases cannot be perfectly matched. Thus, it is necessary to evaluate the commensurability of the phases. The natural stress generated by the two phases in contact (A/B) is typically measured by the mismatch parameter (ξ).

$$\xi = 1 - \frac{2S_{A/B}}{S_A + S_B} \quad \textbf{(S1)}$$

Here S_A and S_B are the surface area, and $S_{A/B}$ is the overlap surface area. A detailed description of how to obtain the surface area values has been previously reported.[13,14] As illustrated in Table S1, Au(111) and γ-Fe_2O_3(111) surfaces comprises the lower value for mismatch displacement (~3.4 %). Because of that Au(111)/γ-Fe_2O_3(111) was selected as a representative interface for the calculations.

Table S1: Mismatch parameter values (ξ) for different γ-Fe_2O_3 surfaces with Au(111). ξ Values were calculated as indicated in Equation S1. ***a***, ***b***, **c**, α, β, and γ are the surface structure parameters obtained using ASE[6] python package. S_A and S_B are the surface area, and $S_{A/B}$ is the overlap surface area.

Surfaces		α, °	β, °	γ, °	**a**, Å	**b**, Å	S_A, Å^2	S_B, Å^2	$S_{A/B}$, Å^2	ξ, %
Au	**(1 1 1)**	**90.00**	**90.00**	**60.00**	**2.89**	**2.89**	---	---	---	---
	**(0 0 1)**	**90.00**	**90.00**	**90.00**	**8.33**	**8.33**	**64.87**	**69.39**	**64.87**	**3.4**
	(2 1 0)	90.00	90.00	90.00	18.63	8.33	129.75	155.16	129.75	8.9
	(2 4 6)	90.00	90.00	36.81	28.86	30.03	720.81	519.26	519.26	16.3
γ-Fe2O3	(1 1 4)	90.00	90.00	35.00	11.78	34.35	345.99	232.07	232.07	19.7
	(1 1 8)	90.00	90.00	35.00	11.78	67.16	663.15	453.79	453.79	18.7
	(2 1 3)	90.00	90.00	35.00	18.63	26.34	389.24	281.43	281.43	16.1
	(1 1 10)	90.00	90.00	35.00	11.78	83.72	836.14	565.66	565.66	19.3

To compute the reaction energies at the interface formed between γ-Fe_2O_3 substrate and Au nanoparticle, we have constructed the Au nanoparticle as a nanowire structure. The Au/γ-Fe_2O_3 interface was designed in two steps: (i) constructing the interface by stacking Au(3×3×1)-fcc(111) and γ-Fe_2O_3(1×2×1)-Fd3m(111) surface structure, and (ii) removing Au atoms to form the structure in Figure S1. As a consequence of step (ii), the obtained structure keeps the periodicity of Au(111) along **a** direction, while the periodicity is removed along **b** direction. A similar procedure has been applied for MoO_2/Pt composites.[15]

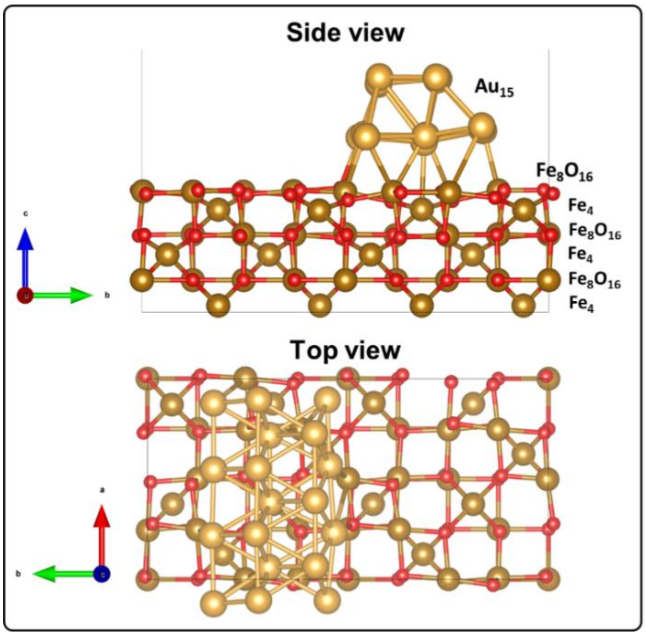

Figure S1: Optimized Au(111)/γ-Fe_2O_3(111) interface structure. Final structure details: {Number of atoms: 99, Chemical formula: $Fe_{36}O_{48}Au_{15}$, Cell parameters: {**a**= 8.48 Å; **b**= 16.96 Å}, vacuum space: 20 Å}. This structure was used to calculate the reaction energetics shown in Figures S2, S3 and S5, and in Table S2.

S2.2 Free-energy calculations

Reaction free energies (ΔG) were calculated based on the equation below:

$$\Delta G = G_{Inter+Au/\gamma-Fe_2O_3} + \sum G_{product\ molecules} - \sum G_{reactant\ molecules} - G_{Au/\gamma-Fe_2O_3} \quad \textbf{(S2)}$$

where $G_{Inter+Au/\gamma-Fe_2O_3}$ and $G_{Au/\gamma-Fe_2O_3}$ are the energies of the surface with and without the adsorbed reaction intermediates, respectively. The sum of reactant/product gas phase molecules (H_2O, O_2, CO, and CO_2) is defined as:

$$\sum G_{reactant\ molecules} = x_r G_{H_2O} + y_r G_{O_2} + z_r G_{CO} \quad \textbf{(S3)}$$

$$\sum G_{product\ molecules} = x_p G_{H_2O} + y_p G_{CO_2} \quad \textbf{(S4)}$$

Here the values {x_r, y_r, and z_r} are the number of reacting molecules and the values {x_p, and y_p} are the number of product molecules. Note that water is in both the reactant and product, since water is not present in the global reaction, "2CO(g) + O_2(g) → 2 CO_2 (g)".

Thermal corrections to free energies were obtained from the frequency analysis by assuming harmonic vibrations. Vibrational contributions were included for the surface states allowing only the adsorbates to vibrate. $G_{Inter+Au/\gamma-Fe_2O_3}$ and $G_{Au/\gamma-Fe_2O_3}$, Gibbs free energies were approximated by the sum of the internal DFT/PBE energy (E) and zero-point energy (ZPE), e.g., $G_{Au/\gamma-Fe_2O_3} = E_{Au/\gamma-Fe_2O_3} + ZPE_{Au/\gamma-Fe_2O_3}$.

The free energy of the molecules ($G_{molecule}$) were calculated as

$$G_{molecule} = E_{molecule} + ZPE_{molecule} + \Delta\mu^0_{molecule}(T^0, p^0) + k_B T\, ln\left(\frac{p^{molecule}}{p^0}\right) \quad \textbf{(S5)}$$

where $\Delta\mu^0_{molecule}(T^0, p^0)$ represents the chemical potential of the molecules at standard conditions, i.e., p^0= 1 bar and T^0=298.15 K. All the values for $\Delta\mu^0_{molecule}(T^0, p^0)$ were obtained in NIST-JANAF tables,[16] and the values for H_2O, O_2, CO, and CO_2 are 0.48, 0.54, 0.53, 0.58 eV, respectively. To combine theoretical calculations with the experiments performed in the present work, the partial pressures of the molecules ($p^{molecule}$) were considered equal to 0.028, 0.200, 0.010 and 0.010 bars for H_2O, O_2, CO, and CO_2, respectively. The DFT calculations predict the reaction free energy for "2CO(g) + O_2(g) → 2 CO_2 (g)" to -6.07 eV, which is in reasonable agreement with previous DFT computations[17] and with tabulated[18] experimental values (-5.3 eV).

S2.3 DFT reaction energetics

Table S2. Summary of DFT calculated reaction energetics for our proposed w-MvK mechanism and for previously proposed LH mechanisms by Chandler[19] and Iglesia.[2] Every reaction elementary step is shown in Figures S2-S5.

Mechanism	Label	Reaction	Total ΔrG, eV	Maximum Ea, eV
Chandler	R1	2 x (CO (g) + * $\rightarrow$ *CO)	-0.42	Adsorption
	R2	2 x (H_2O (g) + ‡ $\rightarrow$ $H_2O^{\ddagger}$)	-0.22	Adsorption
	R3	$H_2O^{\ddagger}$ + O_2 (g) + † $\rightarrow$ †OOH + $^{\ddagger}OH^{-}$	-0.83	0.55
	R4	†OOH + *CO $\rightarrow$ *COOH + $O^{\dagger}$	-1.53	0.31
	R5	*COOH + $H_2O^{\ddagger}$ $\rightarrow$ * + CO_2 (g) + $H_3O^{+\,\ddagger}$	-1.99	0.08
	R6	$O^{\dagger}$ + *CO $\rightarrow$ † + * + CO_2 (g)	-1.26	0.16
	R7	$^{\ddagger}OH^{-}$ + $H_3O^{+\,\ddagger}$ $\rightarrow$ 2 H_2O (g) + 2 ‡	0.18	Desorption
	Total	2 CO(g) + O_2(g) $\rightarrow$ 2 CO_2 (g)	-6.07	0.55
Iglesia Au-γ-Fe_2O_3 interface	R8	2 x (CO (g) + * $\rightarrow$ *CO)	-0.42	Adsorption
	R9	O_2 (g) + * $\rightarrow$ *O_2	-1.02	Adsorption
	R10	H_2O (g) + * $\rightarrow$ H_2O*	-0.70	Adsorption
	R11	*O_2 + H_2O* $\rightarrow$ *OOH + *OH	0.47	0.55
	R12	*OOH + *CO $\rightarrow$ *OH + CO_2 (g) + *	-3.33	0.42
	R13	*OH + *OH $\rightarrow$ H_2O (g) + O*	0.62	0.62
	R14	*CO + O* $\rightarrow$ CO_2 (g) + 2*	-1.69	0.16
	Global	2 CO(g) + O_2(g) $\rightarrow$ 2 CO_2 (g)	-6.07	0.62
Iglesia Au(111)	R8	2 x (CO (g) + * $\rightarrow$ *CO)	0.60	Adsorption
	R9	O_2 (g) + * $\rightarrow$ *O_2	0.99	Adsorption
	R10	H_2O (g) + * $\rightarrow$ H_2O*	0.13	Adsorption
	R11	*O_2 + H_2O* $\rightarrow$ *OOH + *OH	0.04	---
	R12	*OOH + *CO $\rightarrow$ *OH + CO_2 (g) + *	-3.76	---
	R13	*OH + *OH $\rightarrow$ H_2O (g) + O*	-0.58	---
	R14	*CO + O* $\rightarrow$ CO_2 (g) + 2*	-3.49	---
	Global	2 CO(g) + O_2(g) $\rightarrow$ 2 CO_2 (g)	-6.07	Adsorption
w-MvK	R15	2 x (CO (g) + * $\rightarrow$ *CO)	-0.70	Adsorption
	R17	H_2O (g) + O_{lat} $\rightarrow$ OH_{ad} + OH_{lat}	-0.61	0.44
	R18	*CO + OH_{ad} $\rightarrow$ *COOH	0.09	0.31
	R19	*COOH + OH_{lat} $\rightarrow$ CO_2 (g) + H_2O (g) + * + $\square_{O_{lat}}$	0.01	0.36
	R20	O_2 (g) + $\square_{O_{lat}}$ + * $\rightarrow$ O_{lat} + O*	-3.18	Adsorption
	R21	O* + *CO $\rightarrow$ CO_2 (g) + 2 *	-1.68	0.16
	Global	2 CO(g) + O_2(g) $\rightarrow$ 2 CO_2 (g)	-6.07	0.44

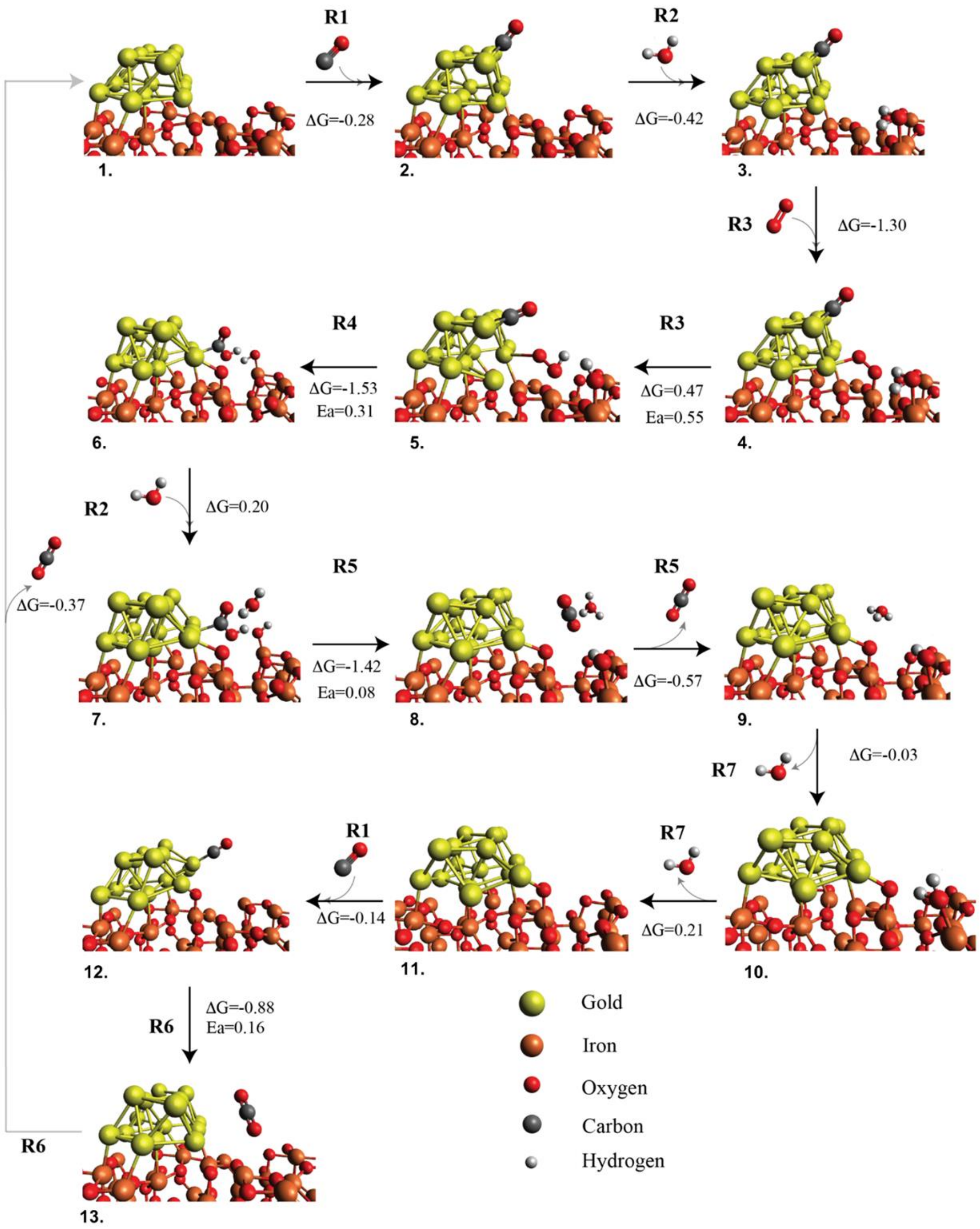

Figure S2. Elementary reactions used for DFT calculation of reaction energetics for Chandler's proposed LH mechanism[19] (Scheme 1, main paper and Table S2). Note that some reactions (R3, R5, R7) were divided into several elementary steps, to enable DFT computation. Reaction free energies and activation free energies in eV. Structures labelled **1. – 13.**

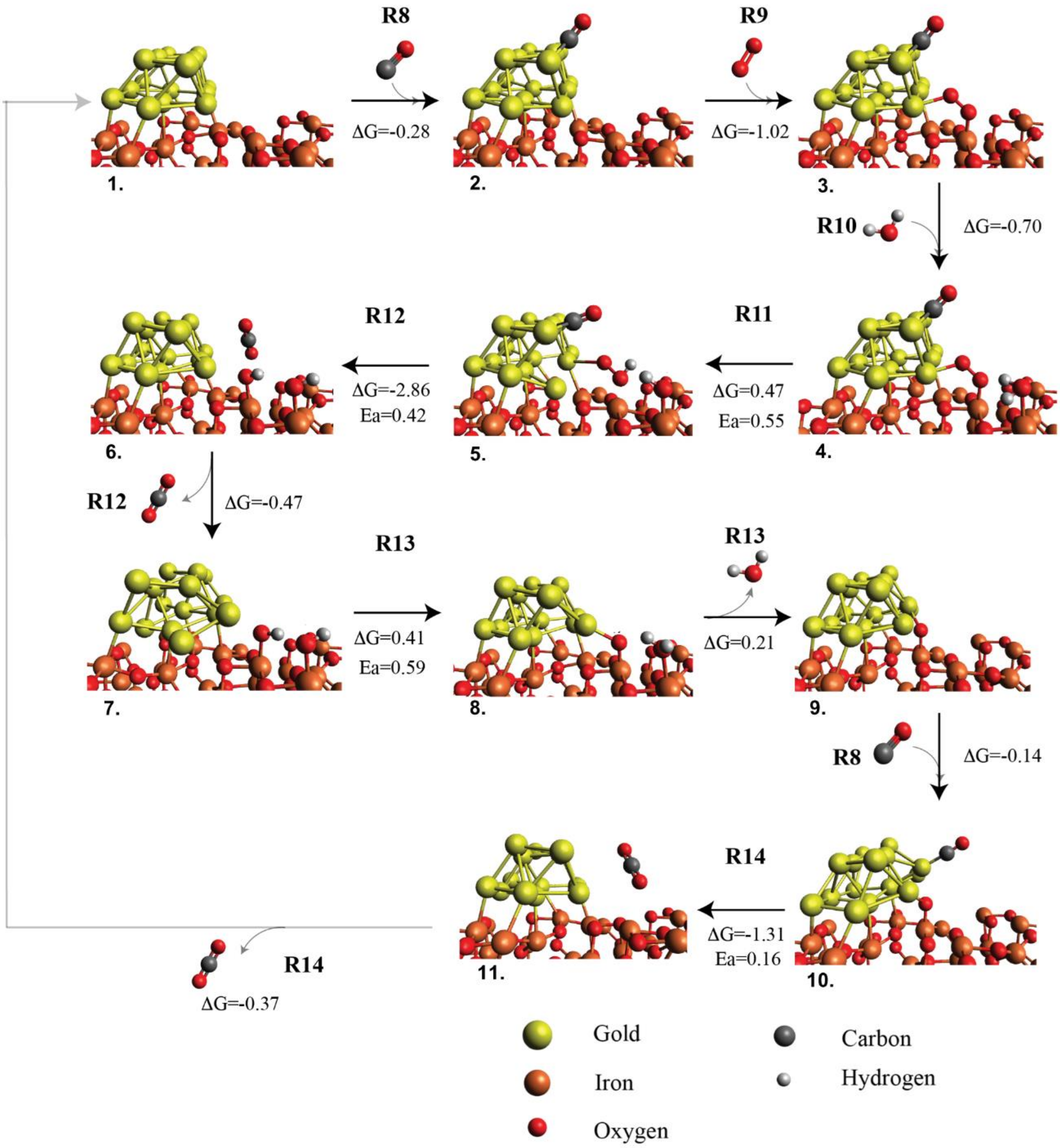

Figure S3. Elementary reactions used for DFT calculation of reaction energetics for Iglesia's proposed LH mechanism[2] (Scheme 2, main paper and Table S2). Note that we allowed this reaction to occur at the Au-γ-Fe_2O_3 interface, (as opposed to exclusively on the Au NP, as proposed by Iglesia). The reason is that the reaction on a pure gold surface is not feasible, since the adsorption free energy for O_2 adsorption on Au(111) was calculated to 0.99 eV (R9, Figure S4 and Table S2). Note that some reactions (R12, R13, R14) were divided into several elementary steps, to enable DFT computation. Reaction free energies and activation free energies in eV. Structures labelled **1. – 11.**

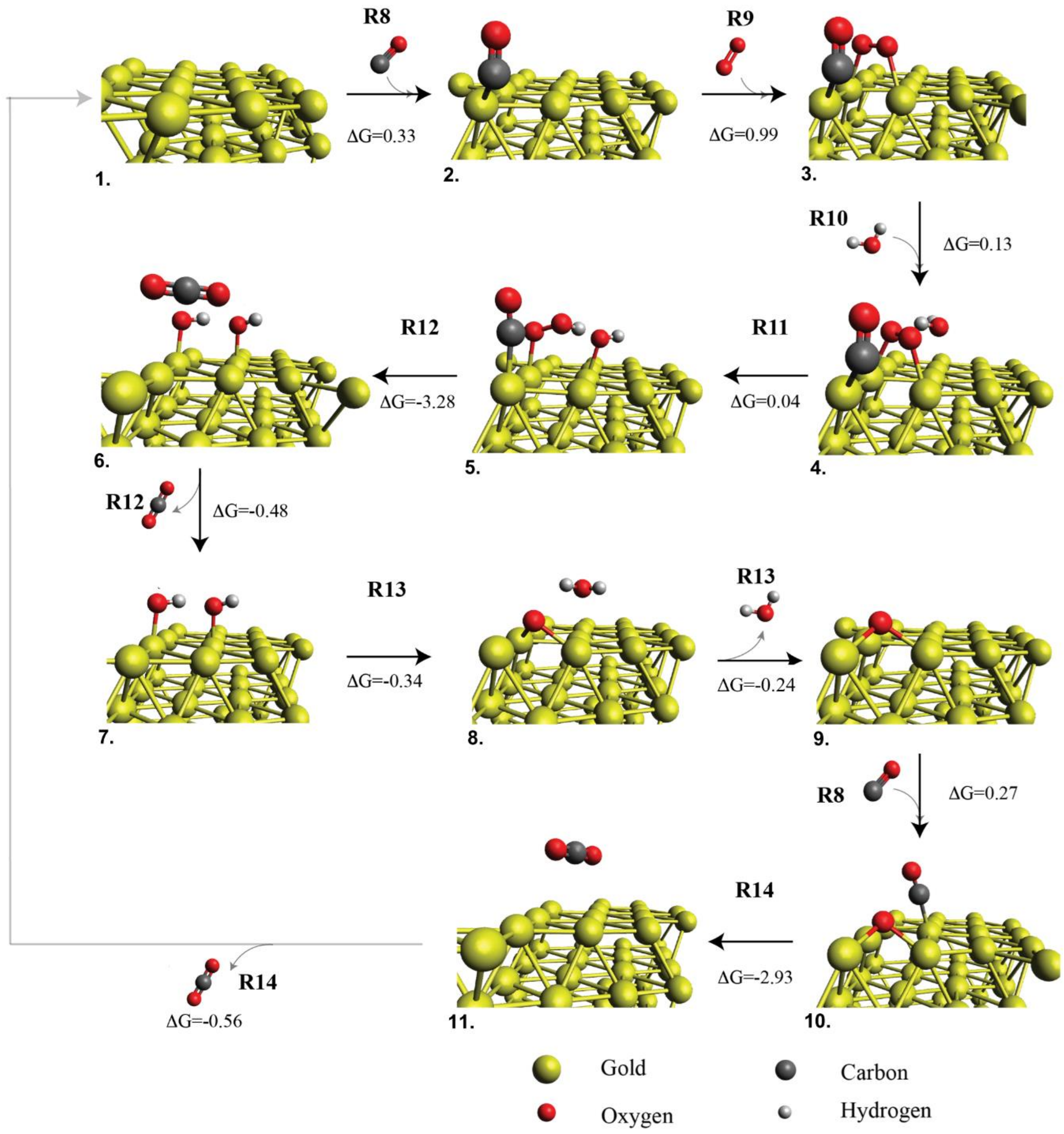

Figure S4. Elementary reactions used for DFT calculation of reaction energetics for Iglesia's proposed LH mechanism[2] (Scheme 2, main paper and Table S2), occurring on Au(111). Note that some reactions (R12, R13, R14) were divided into several elementary steps, to enable DFT computation. . Reaction free energies and activation free energies in eV. Structures labelled **1. – 11.**

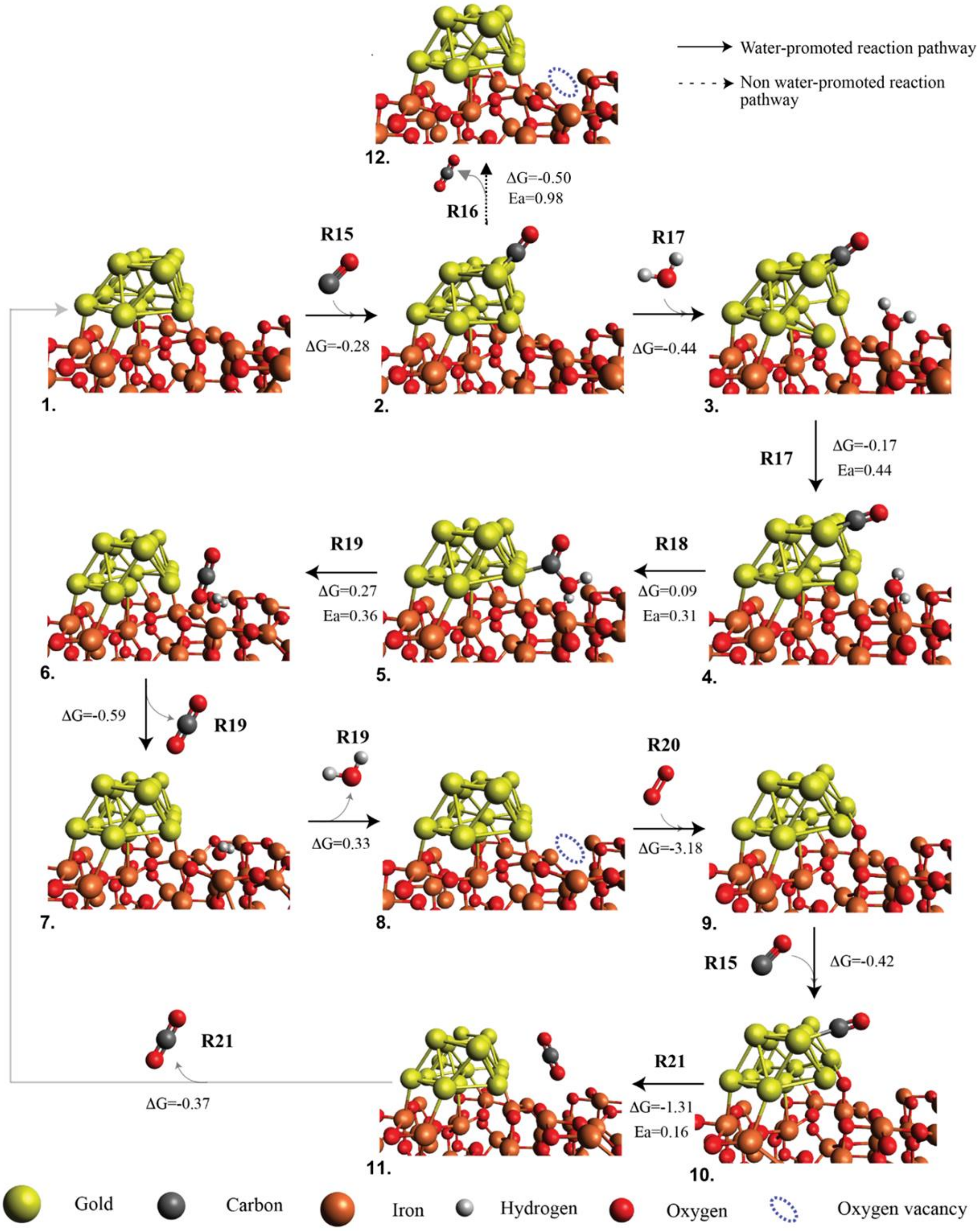

Figure S5. Elementary reactions used for DFT calculation of the reaction energetics (summarized in Table S2) for our proposed w-MvK mechanism of water-promoted CO oxidation over Au-γ-Fe_2O_3. The mechanism is also discussed in detail in the main paper, (Figure 3 and Scheme 3) and is represented by R15, R17 – R21. Direct abstraction of lattice oxygen by CO (non-water promoted) is represented by R16. Reaction free energies and activation free energies in eV. Note that some reactions (R17, R19, R21) were divided into several elementary steps, to enable DFT computation. Reaction free energies and activation free energies in eV. Structures labelled **1. – 12.**

S3. Miscellaneous figures (Figure S5 – Figure S11)

A

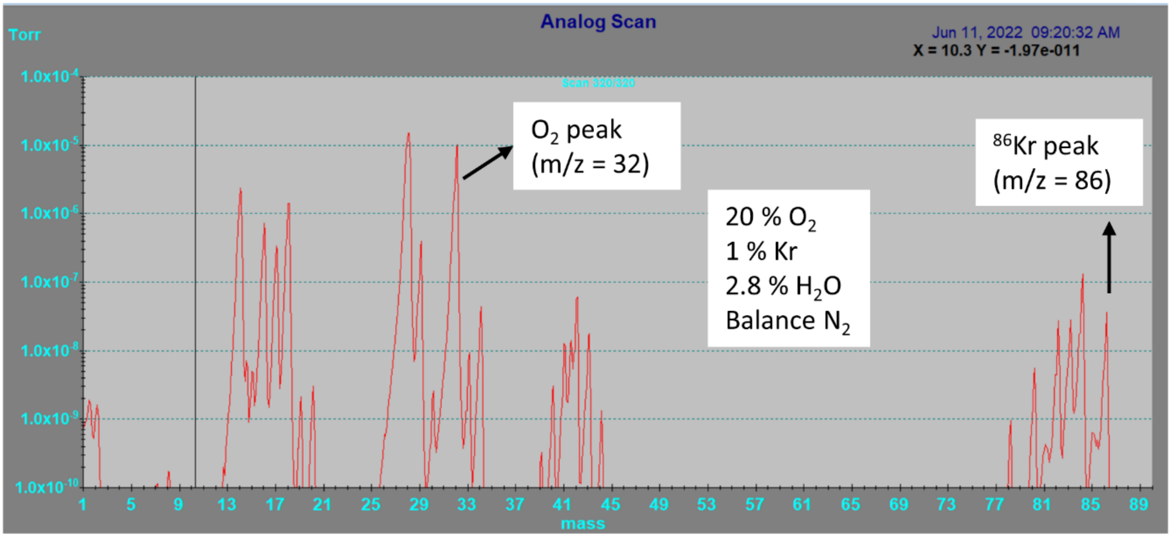

Normalized O_2 peak = Area(m/z=32) / Area(m/z=86) = 350

→ Sensitivity = 350 / 20 % = 17.5 a.u. / % O2 = 0.00175 a.u. / ppm O2

B

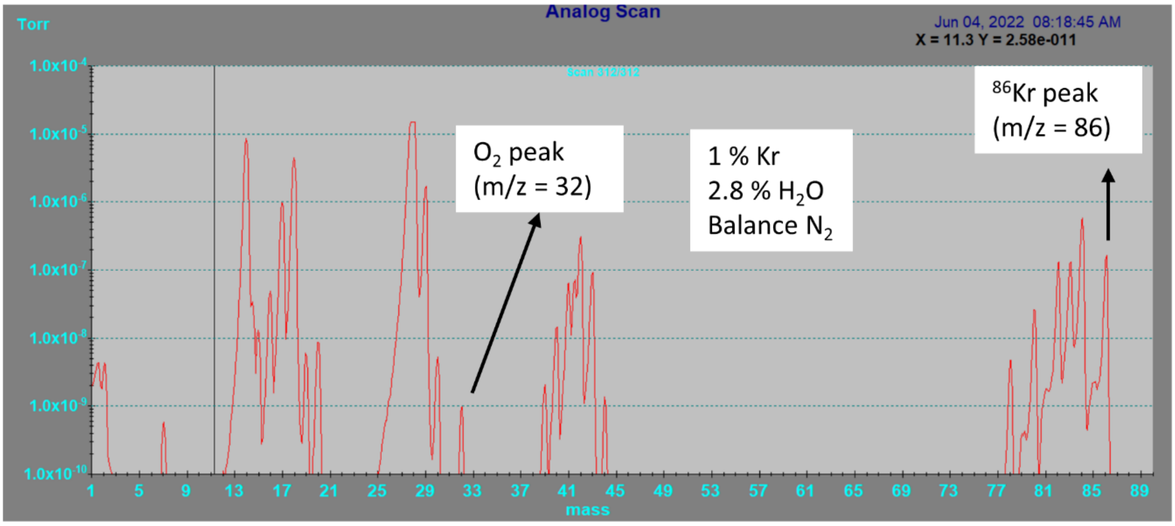

Normalized O_2 peak = Area(m/z=32) / Area(m/z=86) = 0.0065 a.u.

→ Approximate O2 background (%): (0.0065 a.u.) / (0.00175 a.u. / ppm O2) = 4 ppm O2

Figure S6. Estimation of background concentration of O_2 in our reactor. The sensitivity of the mass spectrometer (CIS300, SRS) to O_2 was estimated with a feed gas of 20 vol % O_2, 1 vol % Kr, 2.8 vol % H_2O, balance N_2, flowrate of 14 mL min^{-1}. The sensitivity was 0.00175 a.u. / ppm O_2; typical mass-spectrum shown in **(A)**. The background concentration of O_2 in the reactor under nominally oxygen free conditions (typical mass spectrum in **B**) can then be estimated to 4 ppm. This O_2 background concentration should be compared to the concentration of CO_2 resulting from CO oxidation under nominally O_2-free conditions, which is about 200 ppm (See Figure S7 for an estimation of this concentration). Therefore, the background O_2 concentration in our reactor is about 2 % of the CO_2 concentration during CO oxidation over Au-γ-Fe_2O_3 in nominally O_2-free conditions (Figure 2 B).

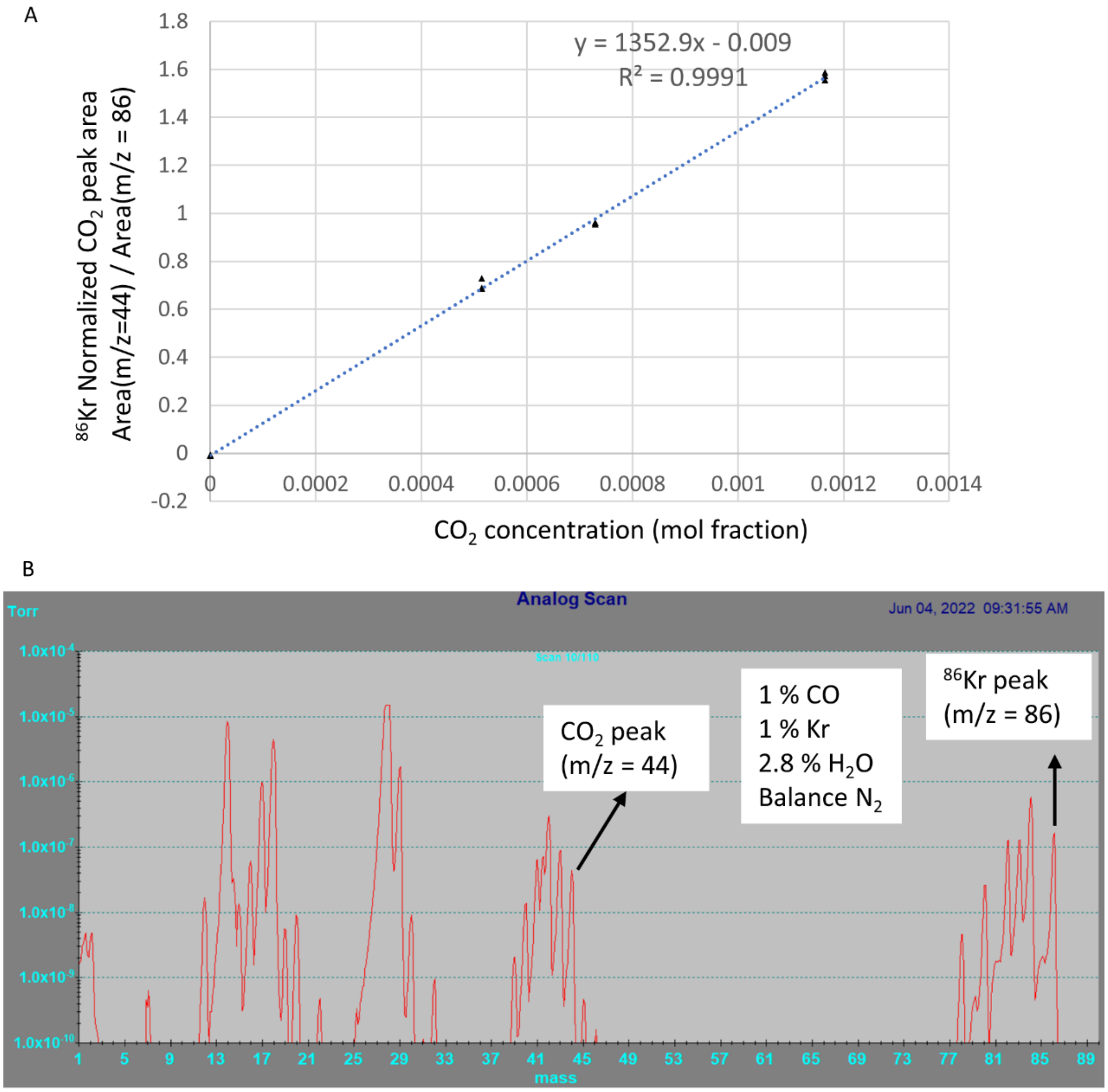

Normalized CO_2 peak = Area(m/z=44) / Area(m/z=86) = 0.25 a.u.

→ CO_2 concentration (mol fraction): (0.25 + 0.009)/1352.9 = 0.00019 = 190 ppm

Figure S7. (A) Base-line corrected calibration curve for CO_2 concentration in our reactor, as measured by mass spectrometry. **(B)** Typical mass-spectrum during measurement of CO oxidation rate over Au-γ-Fe_2O_3 in absence of O_2 (see Figure 2 B for rate data). The concentration of CO_2 measured in this spectrum is (using the calibration curve in (A)) 190 +/- 2 ppm (95 % confidence).

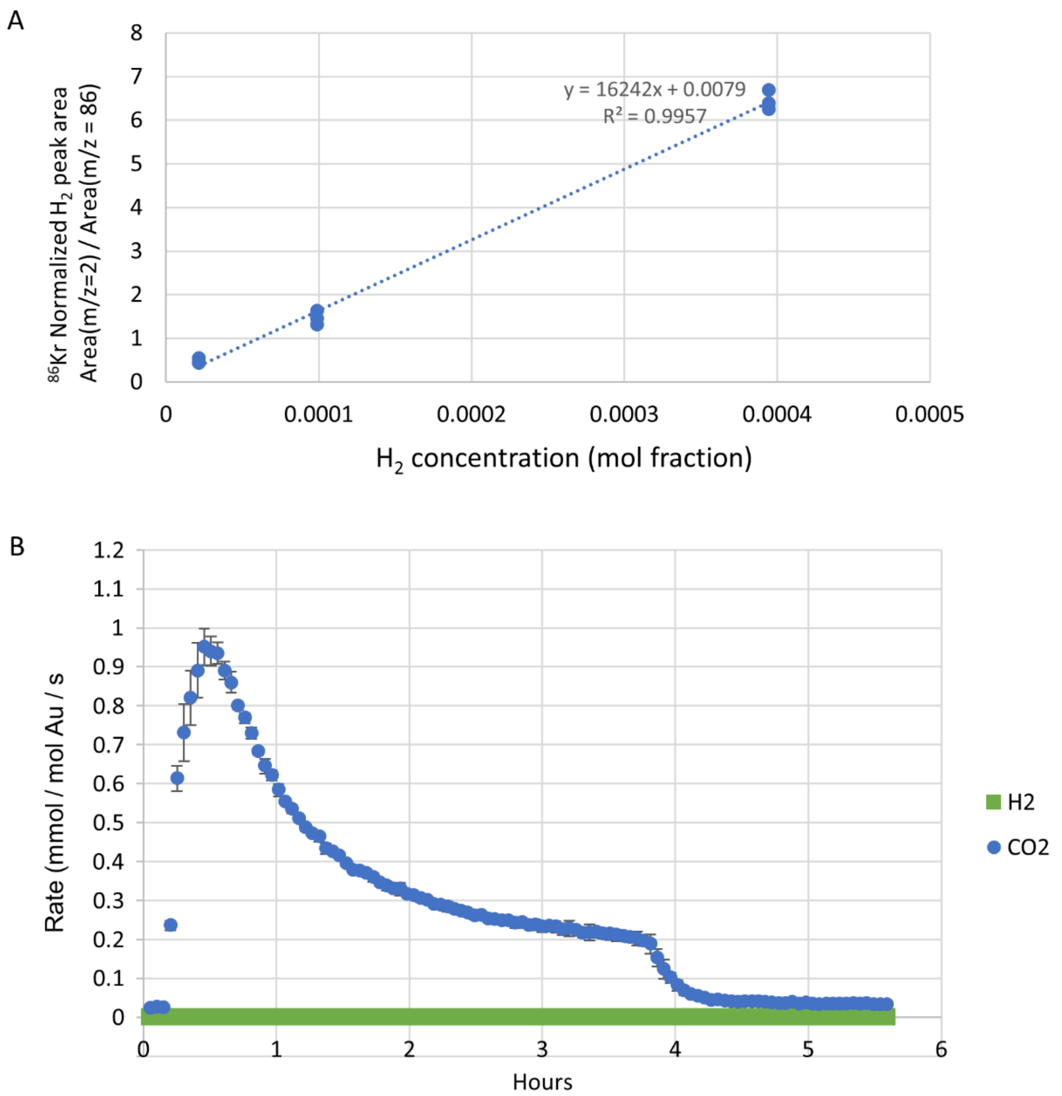

Figure S8. (A) Base-line corrected calibration curve for H_2 concentration in our reactor, as measured by mass spectrometry. **(B)** Transient CO_2 (blue circles) and H_2 (green squares) production rates over Au-γ-Fe_2O_3 during CO oxidation in absence of O_2. Reaction conditions: 1 vol % CO, 2.8 vol % H_2O, balance N_2. Reaction temperature was 25 ºC, and pressure 1 atm. The gas hourly space velocity (GHSV) was 21 L h^{-1} g^{-1}_{cat}, and the CO conversion was below 20 % (ensuring data was collected under differential conditions).[2,3] Note that the CO_2 rate data is the same as the data presented in Figure 2 (B), main paper. Here the CO_2 production is compared with the concomitant H_2 production, which is negligible (therefore ruling out that the CO_2 production is due to the water-gas shift reaction). Reported curves are averages of three independent measurements. Error bars are 2 standard deviations wide. For some data points, the error-bars are so small, they are obscured by the data-labels.

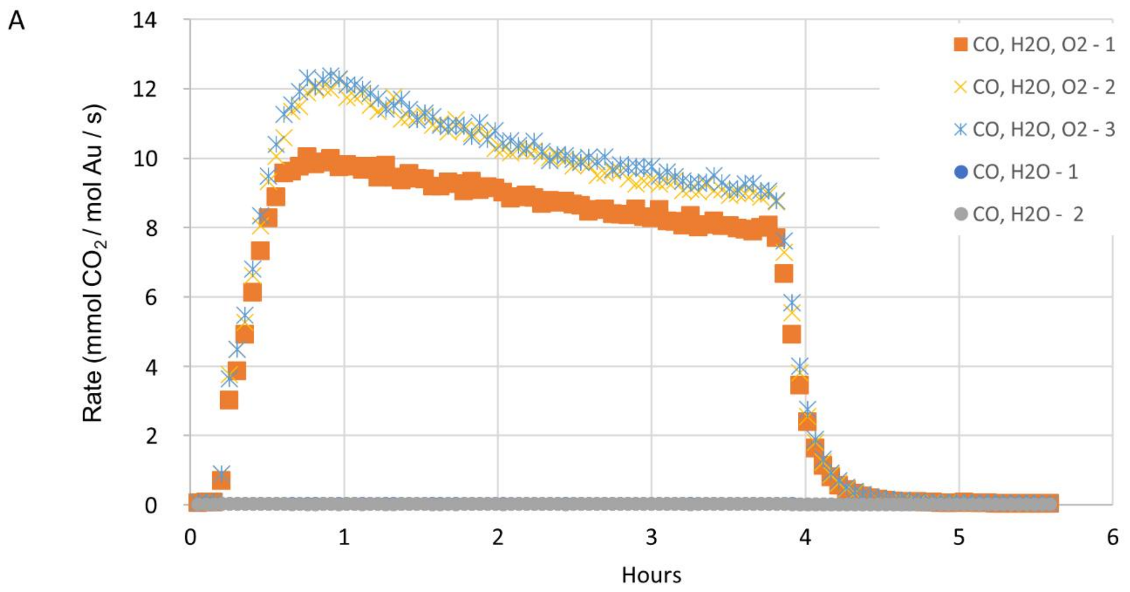

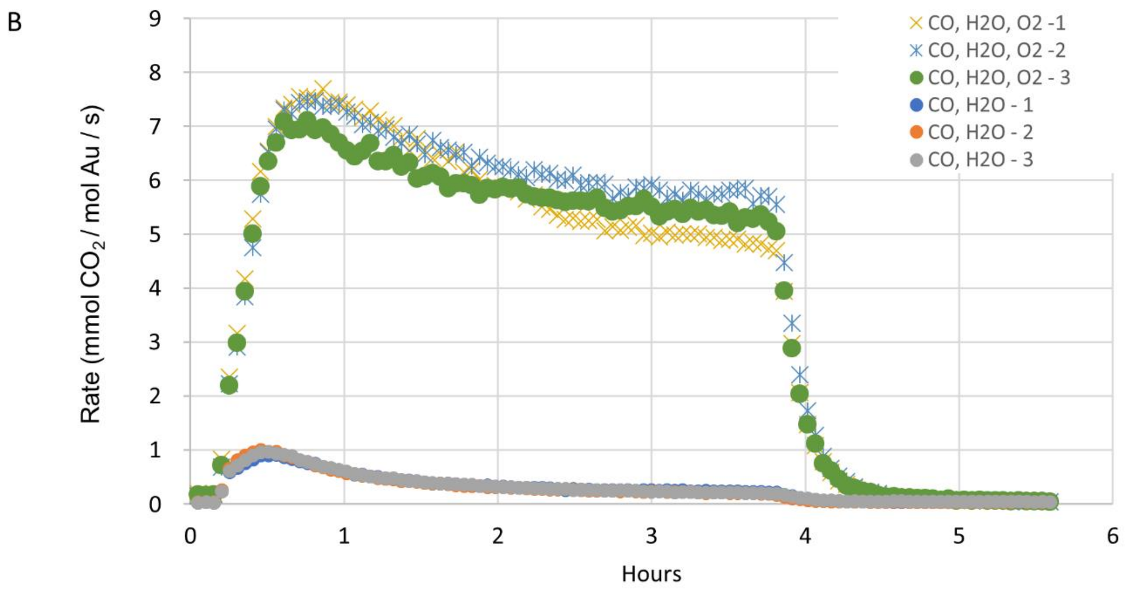

Figure S9. (A) Full data-sets for Figure 2 A, main paper, describing transient CO oxidation rates over Au-TiO_2. **(B)** Full data-sets for Figure 2 B, main paper, describing transient CO oxidation rates over Au-γ-Fe_2O_3. Refer to Figure 2 for reaction conditions. Refer to Figure S7 A for the CO_2 calibration used to determine CO_2 concentrations in the reactor effluent. Note that for Au-TiO_2, no significant CO_2 production was observed in absence of O_2, and therefore this measurement was only repeated two times.

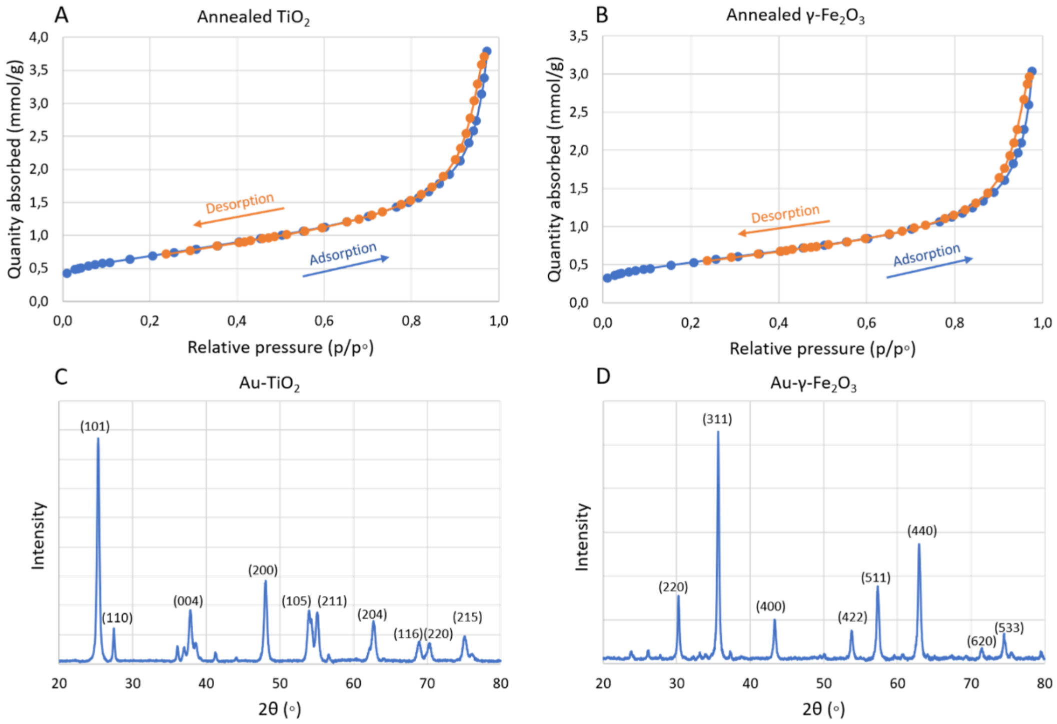

Figure S10. Nitrogen adsorption isotherm plots for **A)** TiO_2 and **B)** γ-Fe_2O_3. Brunauer-Emmet-Teller (BET) analysis yields a surface area of 54.8 m^2/g for TiO_2 and 42.5 m^2/g for γ-Fe_2O_3. Powder X-ray diffraction patterns of **C)** Au-TiO_2, with peak assignments (JCPDS-card 21-1272 and JCPDS card 21-1276) for anatase and rutile TiO_2, and **D)** Au-γ-Fe_2O_3, with peak assignments (JCPDS-card 39-1346) for γ-Fe_2O_3.

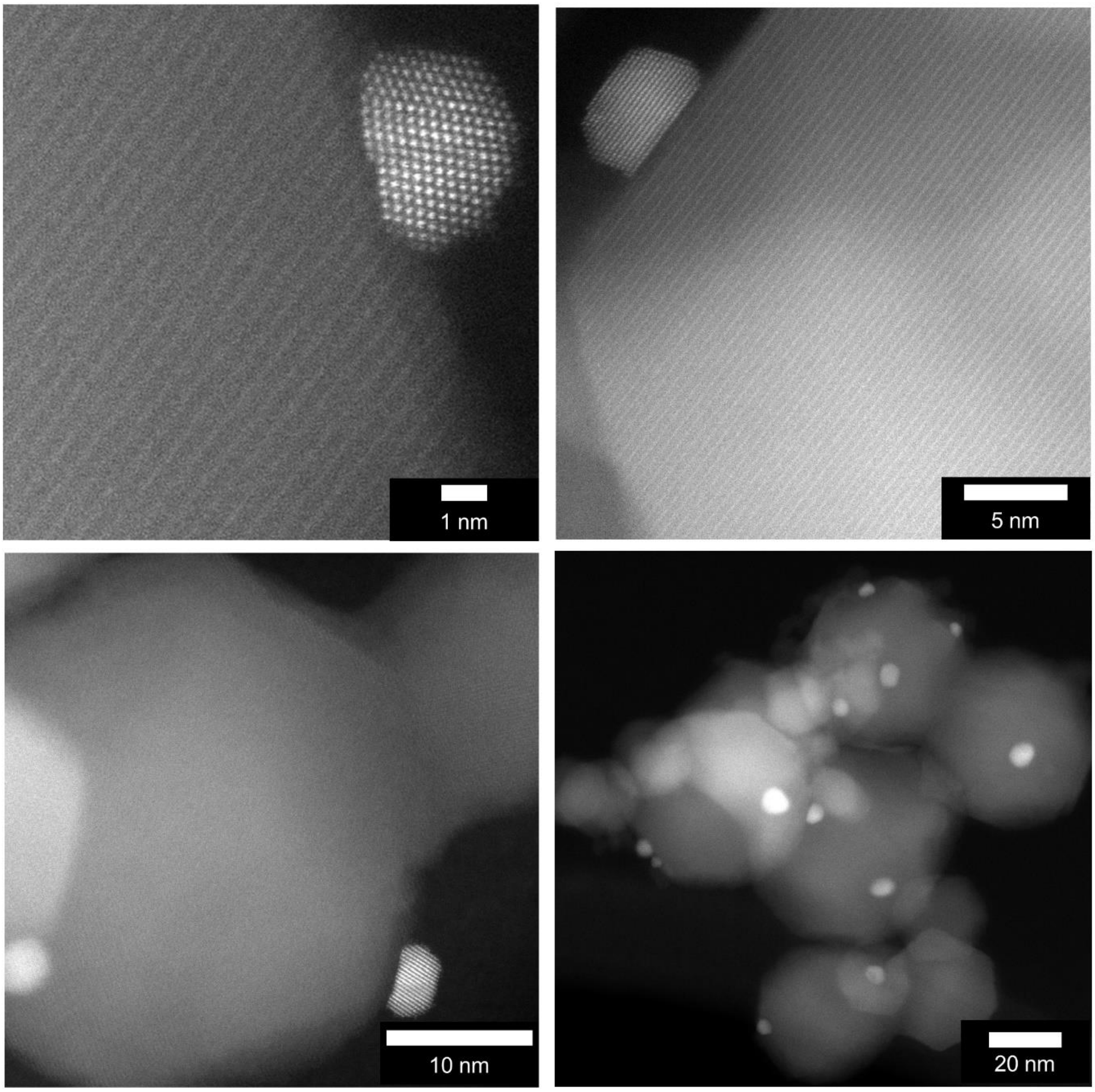

Figure S11. Examples of HAADF-STEM micrographs of the Au-γ-Fe_2O_3 catalyst. After investigation of many (>20) such micrographs, we could not find any evidence of single Au atoms, or very small Au-clusters. We therefore conclude that Au NPs in the range 2 – 8 nm (Figure 1 D) are the dominating Au-species in the catalyst.

S4. Calculation of predicted abundances of $C^{16}O_2$, $C^{16}O^{18}O$ and $C^{18}O_2$ resulting from $C^{16}O$ oxidation with $^{16}O_2$ and $H_2{}^{18}O$ over Au-γ-Fe_2O_3.

In the presence of $^{16}O_2$ and $H_2{}^{18}O$ the previously proposed LH-mechanisms and our proposed w-MvK mechanism are expected to lead to different abundances of $C^{16}O_2$, $C^{16}O^{18}O$ and $C^{18}O_2$. To calculate the abundances predicted by these mechanisms, first we must consider that all CO_2 species ($C^{16}O_2$, $C^{16}O^{18}O$ and $C^{18}O_2$) exchange oxygen directly with $H_2{}^{18}O$ in our reactor set-up. Then, we must separate this exchange from the ^{18}O incorporation from $H_2{}^{18}O$ into CO_2 due to the CO oxidation reaction mechanism.

To estimate the direct ^{18}O exchange between $H_2{}^{18}O$ and different isotopic CO_2 species, we first mixed $C^{16}O_2$ (0.34 mol %), $H_2{}^{18}O$ (2.8 mol %) and $^{16}O_2$ (20 mol %), balance N_2, and then passed this mixture over the Au-γ-Fe_2O_3 catalyst, at a flow-rate (8.7 mL min^{-1}) which is similar to the flow-rate (7 mL min^{-1}) used for $C^{16}O$ oxidation with $H_2{}^{18}O$ and $^{16}O_2$ (Figure 5). The slightly higher flowrate in the control experiment was used to allow the $C^{16}O_2$ concentration in the control to correspond to the maximum total CO_2 concentration obtained during $C^{16}O$ oxidation with $H_2{}^{18}O$ and $^{16}O_2$. The time evolution of the relative fractions (%) of $C^{16}O_2$, $C^{16}O^{18}O$ and $C^{18}O_2$ in the reactor effluent, are presented in Figure S12. At time t = 0 h the flow was turned from bypassing the catalyst bed, to pass the catalyst bed. At this time, the relative abundances are 93.2 % ($C^{16}O_2$), 6.3 % $C^{16}O^{18}O$ and 0.4 % ($C^{18}O_2$), suggesting there is some ^{18}O exchange from $H_2{}^{18}O$ into $C^{16}O_2$ in the piping. Upon turning the flow over the catalyst bed, new steady state relative ratios are established, namely 57.5 % ($C^{16}O_2$), 35.9 % ($C^{16}O^{18}O$) and 6.6 % ($C^{18}O_2$). These steady-state abundances can be interpreted as a property of the reactor/catalyst-bed set-up, and can be translated into the probability that a $C^{16}O_2$ molecule transforms either to $C^{16}O^{18}O$ or $C^{18}O_2$ by single (Equation S6) or double ^{18}O (Equation S7) exchange with $H_2{}^{18}O$:

$$P_{exch.}(C^{16}O_2 \longrightarrow C^{16}O^{18}O) = 0.359 \quad \textbf{(S6)}$$

$$P_{exch.}(C^{16}O_2 \longrightarrow C^{18}O_2) = 0.066 \quad \textbf{(S7)}$$

From these equations we can also derive the probability that a $C^{16}O^{18}O$ molecule transforms into $C^{18}O_2$ in the reactor/catalyst-bed set-up. First, we note that a single ^{18}O exchange leads to $C^{18}O_2$ with half the probability given by equation S6 (0.359/2), because in $C^{16}O^{18}O$ half of the exchanges are with the ^{16}O and other half with the ^{18}O. Then, we observe that double ^{18}O exchange in $C^{16}O^{18}O$, which also produces $C^{18}O_2$, should have the same probability as the ^{18}O exchange with both ^{16}O in $C^{16}O_2$, (0.066, equation S7). Therefore, the probability of forming $C^{18}O_2$ by ^{18}O exchange with $C^{16}O^{18}O$ is given by the combined probability of single and double ^{18}O exchanges (Equation S8),

$$P_{exch.}(C^{16}O^{18}O \longrightarrow C^{18}O_2) = 0.359 / 2 + 0.066 = 0.246 \quad \textbf{(S8)}$$

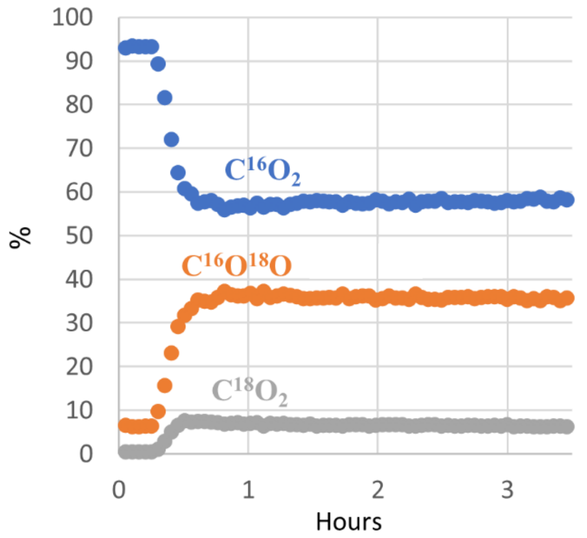

Figure S12. Time evolution of the relative fractions (%) of $C^{16}O_2$, $C^{16}O^{18}O$ and $C^{18}O_2$ in the $C^{16}O_2$ + $H_2{}^{18}O$ control-experiment to determine reactor-specific probability of transformation of different CO_2 – species into other CO_2 – species by ^{18}O exchange with $H_2{}^{18}O$.

Now, let us predict the relative abundances of the CO_2 isotopic species resulting from $C^{16}O$ oxidation with $^{16}O_2$ and $H_2{}^{18}O$ assuming the different reaction mechanisms considered in this paper. First, we consider the LH mechanism proposed by Chandler et. al. (Scheme 1 in main paper). Here we adapt this scheme to describe the reaction involving $C^{16}O$, $^{16}O_2$ and $H_2{}^{18}O$ (Scheme S1). In this mechanism, water only participates via a series of proton exchanges, and no ^{18}O incorporation into CO_2 is expected. As a consequence, Chandler's LH mechanism would result in 100 % $C^{16}O_2$. Next, consider the LH mechanism proposed by Iglesia et al. (scheme 2 in main paper). Here we adapt this scheme to describe the reaction involving $C^{16}O$, $^{16}O_2$ and $H_2{}^{18}O$ (Scheme S2). By studying scheme S2 we conclude that Iglesias's LH mechanism would result in 75 % $C^{16}O_2$ and 25 % $C^{16}O^{18}O$. Finally, in Scheme S3, consider the w-MvK mechanism proposed in this work (Scheme 3 in main paper) adapted to the reaction involving $C^{16}O$, $^{16}O_2$ and $H_2{}^{18}O$. By analyzing Scheme S3, we conclude that our proposed w-MvK mechanism would result in 50 % $C^{16}O_2$ and 50 % $C^{16}O^{18}O$. To further illustrate the CO_2 isotopic abundancies that should be expected from the different mechanisms, consider Figure S13 – Figure S16, where we reproduce Figures S2 – Figure S5, but using $H_2{}^{18}O$ instead of $H_2{}^{16}O$.

We now have knowledge of ^{18}O exchange probabilities between different isotopic CO_2 species and $H_2{}^{18}O$ in our reactor (equations S6 – S8) and we have predicted the abundancies of different isotopic CO_2 species that would result from the previously proposed LH mechanisms[2,19] (Scheme S1, Scheme S2 and Figure S13 – S15) and our proposed w-MvK mechanism (Scheme S3 and Figure S16). Using a simple probability tree (Scheme S4) we can therefore predict which fraction of the different isotopic CO_2 species should be detected in our reactor effluent according to the different mechanisms. Following the probability tree, we predict that if Chandlers's LH mechanism is the dominating reaction mechanism, we should expect 57.5% $C^{16}O_2$, 35.9% $C^{16}O^{18}O$ and 6.6 % $C^{18}O_2$ in the reactor effluent. If Iglesia's LH mechanism is the dominating reaction mechanism, we should expect 43.1 % $C^{16}O_2$, 45.8 % $C^{16}O^{18}O$ and 11.1 % $C^{18}O_2$ in the reactor effluent. Finally, if our proposed w-MvK mechanism is the dominating reaction mechanism we should expect 28.8 % $C^{16}O_2$, 55.7 % $C^{16}O^{18}O$ and 15.6 % $C^{18}O_2$ in the reactor effluent.

We wish to point out that in predicting the abundances expected to result from the different mechanisms, we assume that no other elementary reactions occur other than what is explicitly listed in

the respective mechanisms (Scheme 1 – Scheme 3). We note that for the previously proposed LH mechanisms, there could be scrambling reactions (of ^{16}O and ^{18}O) between different reaction intermediates (such as adsorbed O, CO, O_2, OOH, OH, and H_2O). Combinations of such scrambling reactions could possibly take place so that the predicted isotopic abundance resulting from the respective LH mechanisms would be close to the experimentally observed abundances of 50 % $C^{16}O_2$ and 50 % $C^{16}O^{18}O$. We have carried out additional DFT calculations on some potential ^{18}O exchange pathways that could change the predicted isotopic CO_2 abundances in the LH mechanisms (Table S3). However, none of these potential pathways appear plausible compared to the much lower reaction barriers in the respective LH mechanisms. We therefore believe the predictions (Scheme S1 – Scheme S3) of the isotopic CO_2 abundances for the mechanisms evaluated in this manuscript are robust. Moreover, since the predicted abundance of 50 % $C^{16}O_2$ and 50 % $C^{16}O^{18}O$ emerges from the w-MvK mechanism without making any additional assumptions about scrambling reactions, we believe the w-MvK mechanism offers the most straightforward rationalization of our experimental data.

Scheme S1. Elementary reaction steps for the (by Chandler et al.)[2] postulated LH-reaction mechanism of water-promoted CO oxidation over Au-Al_2O_3, Au-TiO_2, and later postulated over Au-Fe_2O_3.[20]. Here we adapt Scheme 1 (main paper) to consider the reaction with $C^{16}O$, $^{16}O_2$ and $H_2{}^{18}O$. * denotes an active site on the Au NP, away from the NP-support interface, † denotes an Au site at the NP-support interface, and ‡ denotes a support site at the NP-support interface. Also consider Figure S13, for further illustration of isotopic CO_2 abundancies that are expected to result from Chandler's proposed mechanism.

R1.	$2 \text{ x } (C^{16}O\ (g) + * \longrightarrow *C^{16}O)$
R2.	$2 \text{ x } (H_2{}^{18}O\ (g) + {}^{\ddagger} \longrightarrow H_2{}^{18}O^{\ddagger})$
R3.	$H_2{}^{18}O^{\ddagger} + {}^{16}O_2\ (g) + {}^{\dagger} \longrightarrow {}^{\dagger 16}O^{16}OH + {}^{\ddagger 18}OH^{-}$
R4.	${}^{\dagger 16}O^{16}OH + *C^{16}O \longrightarrow *C^{16}O^{16}OH + {}^{16}O^{\dagger}$
R5.	$*C^{16}O^{16}OH + H_2{}^{18}O^{\ddagger} \longrightarrow * + C^{16}O_2\ (g) + H_3{}^{18}O^{+\ \ddagger}$
R6.	${}^{16}O^{\dagger} + *C^{16}O \longrightarrow {}^{\dagger} + * + C^{16}O_2\ (g)$
R7.	${}^{\ddagger 18}OH^{-} + H_3{}^{18}O^{+\ \ddagger} \longrightarrow 2\ H_2{}^{18}O\ (g) + 2\ {}^{\ddagger}$
Σ	$2\ C^{16}O\ (g) + {}^{16}O_2\ (g) \longrightarrow 2\ C^{16}O_2\ (g)$

Scheme S2. Elementary reaction steps for the LH-reaction mechanism postulated by Iglesia et al. of water-promoted CO oxidation over Au-Al_2O_3, Au-TiO_2, and Au-Fe_2O_3.[2] Here we adapt Scheme 2 (main paper) to consider the reaction with $C^{16}O$, $^{16}O_2$ and $H_2{}^{18}O$. Where more than one isotope can react in an elementary step, this has been indicated by the appropriate fraction of the stoichiometric coefficient. After summing up the overall catalytic cycle, the overall reaction has been multiplied with a factor of 2, so that all stochiometric coefficients are whole numbers. * denotes an active site on the Au NP. Also consider Figure S14 and Figure S15, for further illustration of isotopic CO_2 abundancies that are expected to result from Iglesia's proposed mechanism.

R8.	$2 \text{ x } (C^{16}O\ (g) + * \longrightarrow *C^{16}O)$
R9.	${}^{16}O_2\ (g) + * \longrightarrow *{}^{16}O_2$
R10.	$H_2{}^{18}O\ (g) + * \longrightarrow H_2{}^{18}O*$
R11.	$*{}^{16}O_2 + H_2{}^{18}O* \longrightarrow *{}^{16}O^{16}OH + *{}^{18}OH$
R12.	$*{}^{16}O^{16}OH + *C^{16}O \longrightarrow *{}^{16}OH + C^{16}O_2\ (g) + *$
R13.	$*{}^{18}OH + *{}^{16}OH \longrightarrow 0.5\ H_2{}^{16}O\ (g) + 0.5\ H_2{}^{18}O\ (g) + 0.5\ {}^{16}O* + 0.5\ {}^{18}O*$
R14.	$*C^{16}O + 0.5\ {}^{16}O* + 0.5\ {}^{18}O* \longrightarrow 0.5\ C^{16}O_2\ (g) + 0.5\ C^{16}O^{18}O\ (g) + 2*$
2Σ	$4\ C^{16}O\ (g) + 2\ {}^{16}O_2\ (g) + 2\ H_2{}^{18}O\ (g) \longrightarrow 3\ C^{16}O_2\ (g) + C^{16}O^{18}O\ (g) + H_2{}^{16}O\ (g) + H_2{}^{18}O\ (g)$

Scheme S3. Elementary reaction steps for our proposed water-promoted w-MvK mechanism. Here we adapt Scheme 3 in the main paper to consider the reaction with $C^{16}O$, $^{16}O_2$ and $H_2{}^{18}O$. * denotes an active site on the Au NP, O_{lat} denotes a lattice-oxygen, near the Au NP, $\square_{O_{lat}}$ denotes a lattice-oxygen vacancy, OH_{ad} denotes a hydroxyl on a lattice-Fe, OH_{lat} denotes a hydroxyl in an oxygen lattice-position. Also consider Figure S16, for further illustration of isotopic CO_2 abundancies that are expected to result from our proposed w-MvK mechanism.

R15. $2 \times (C^{16}O\ (g) + * \longrightarrow *C^{16}O)$

R17. $H_2{}^{18}O\ (g) + {}^{16}O_{lat} \longrightarrow {}^{18}OH_{ad} + {}^{16}OH_{lat}$

R18. $*C^{16}O + {}^{18}OH_{ad} \longrightarrow *C^{16}O^{18}OH$

R19. $*C^{16}O^{18}OH + {}^{16}OH_{lat} \longrightarrow C^{16}O^{18}O\ (g) + H_2{}^{16}O\ (g) + * + \square_{O_{lat}}$

R20. $^{16}O_2\ (g) + \square_{O_{lat}} + * \longrightarrow {}^{16}O_{lat} + {}^{16}O*$

R21. $^{16}O* + *C^{16}O \longrightarrow C^{16}O_2\ (g) + 2\ *$

Σ $2\ C^{16}O\ (g) + {}^{16}O_2\ (g) + H_2{}^{18}O\ (g) \longrightarrow C^{16}O_2\ (g) + C^{16}O^{18}O\ (g) + H_2{}^{16}O\ (g)$

Scheme S4. Probability-tree describing predicted fractions of the different CO_2 species in the reactor effluent of the $C^{16}O$ oxidation with $^{16}O_2$ and $H_2{}^{18}O$ over Au-γ-Fe_2O_3 with Chandler's LH, Iglesia's LH and our proposed water-promoted w-MvK mechanisms. Each branch represents the probability of forming a specific CO_2 species through the $C^{16}O$ oxidation mechanism followed by ^{18}O exchange with $H_2{}^{18}O$. The expected fraction of a specific CO_2 species is then the sum of the probabilities of forming that species in the reactor. The probability of ^{18}O incorporation (x) through the CO oxidation mechanisms is the ratio of the stoichiometric coefficients of $C^{16}O^{18}O$ and ($C^{16}O_2$+$C^{16}O^{18}O$) in the total reaction of Schemes S1-S3. The probability of no ^{18}O incorporation in the oxidation step is 1-x. The probabilities of ^{18}O exchange between the formed CO_2 species and $H_2{}^{18}O$ are given by equations S6–S8.

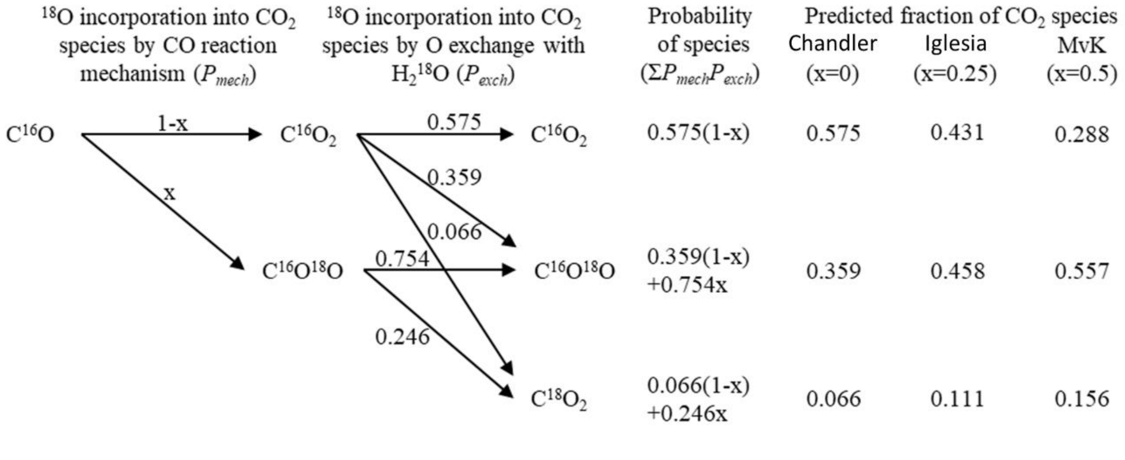

Table S3. Summary of DFT calculated reaction energetics for potential ^{18}O exchange reactions in the three mechanisms studied in this paper (Chandler, Iglesia and our proposed MvK mechanism).

Mechanism	Reaction	ΔrG, eV	Ea, eV
Chandler	$^{\dagger 16}O^{16}OH + {}^{\ddagger 18}OH^- \rightarrow {}^{\dagger 16}O^{18}OH + {}^{\ddagger 16}OH^-$	0	1.01
Chandler	$*C^{16}O^{16}OH + {}^{\ddagger 18}OH^- \rightarrow *C^{16}O^{18}OH + {}^{\ddagger 16}OH^-$	0	0.95
Iglesia	$*^{16}O^{16}OH + *^{18}OH \rightarrow *^{16}O^{18}OH + *^{16}OH$	0	1.01
w-MvK	$^{18}OH_{ad} + {}^{16}OH_{lat} \rightarrow {}^{16}OH_{ad} + {}^{18}OH_{lat}$	0	1.24
w-MvK	$*C^{16}O^{18}OH \rightarrow *C^{18}O^{16}OH$	0	0.79

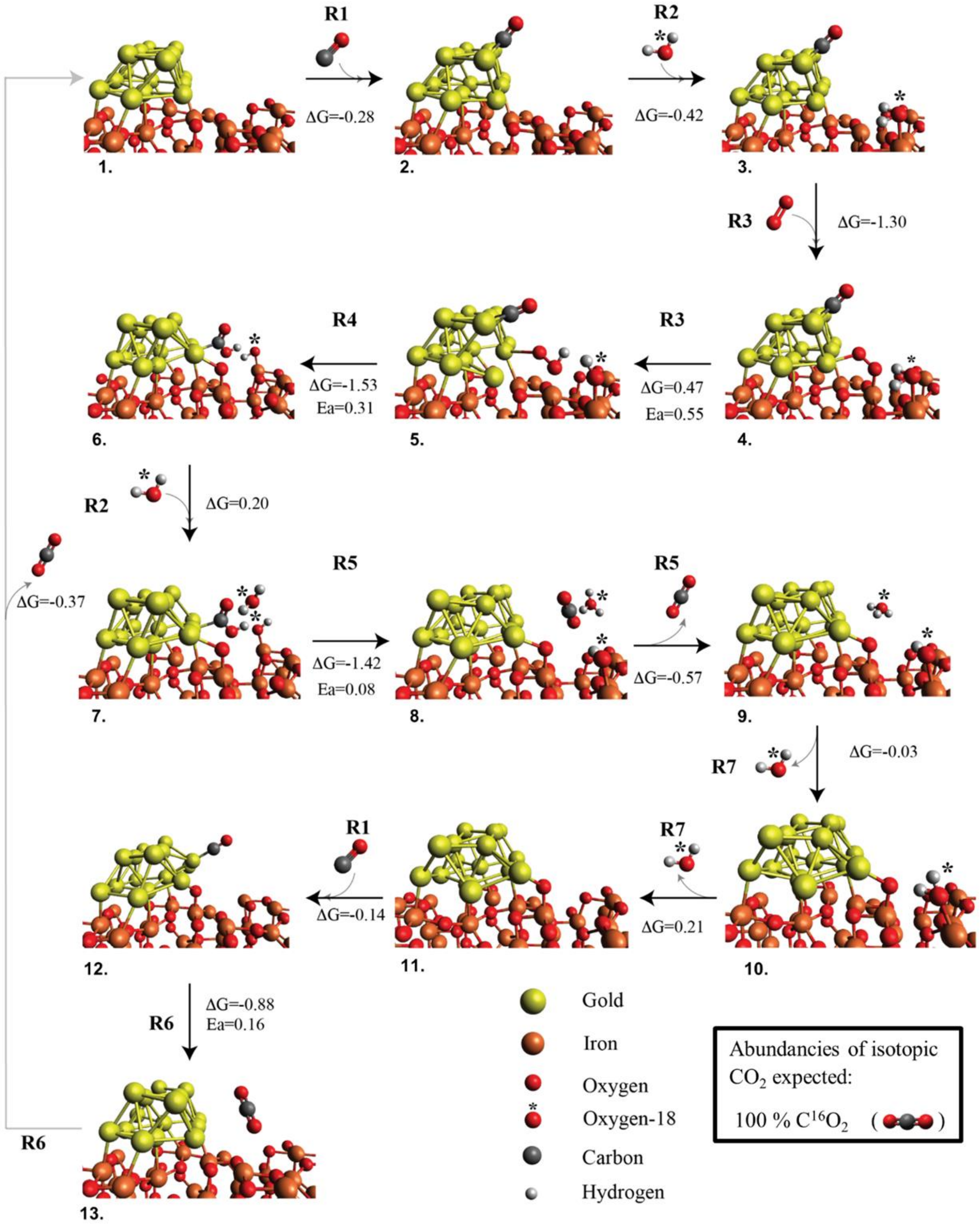

Figure S13. Reproduction of Figure S2, but with $H_2{}^{18}O$ instead of $H_2{}^{16}O$. The figure illustrates how the abundancies of different isotopic CO_2 species can be predicted from Chandler's proposed reaction mechanism[19] (Scheme 1 main paper, and Scheme S1) of water-promoted CO oxidation with $C^{16}O$, $^{16}O_2$ and $H_2{}^{18}O$. It can thus be predicted the mechanism would result in 100 % $C^{16}O_2$. Reaction free energies and activation free energies in eV. Structures labelled **1. – 13.**

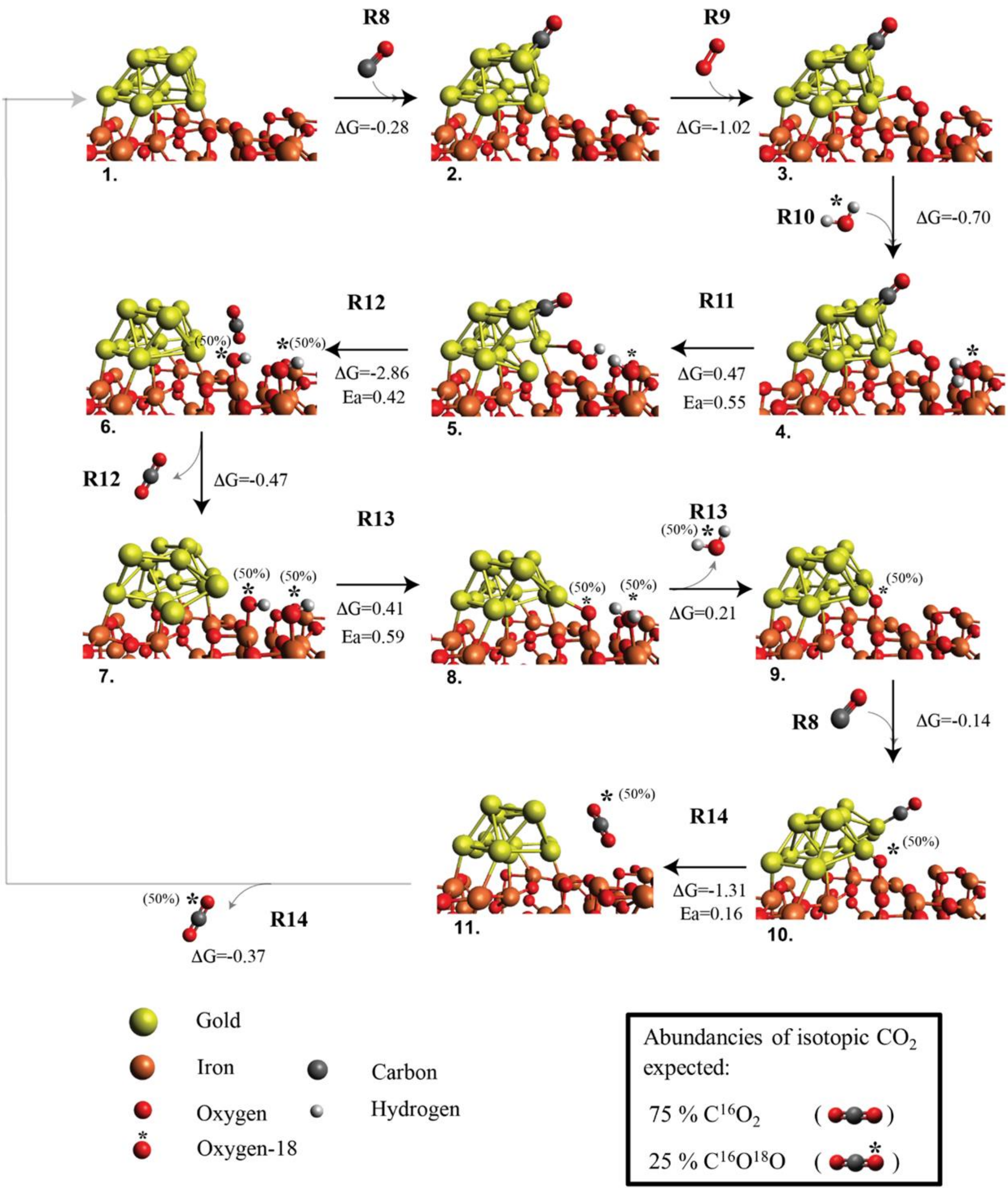

Figure S14. Reproduction of Figure S3, but with $H_2{}^{18}O$ instead of $H_2{}^{16}O$. The figure illustrates how the abundancies of different isotopic CO_2 species can be predicted from Iglesia's proposed reaction mechanism[2] (Scheme 2 main paper, and Scheme S2) of water-promoted CO oxidation with $C^{16}O$, $^{16}O_2$ and $H_2{}^{18}O$. Here illustrated allowing the mechanism to run on both the Au NP and the γ-Fe_2O_3 support. It can thus be predicted the mechanism would result in 75 % $C^{16}O_2$ and 25 % $C^{16}O^{18}O$. Reaction free energies and activation free energies in eV. Structures labelled **1. – 11.**

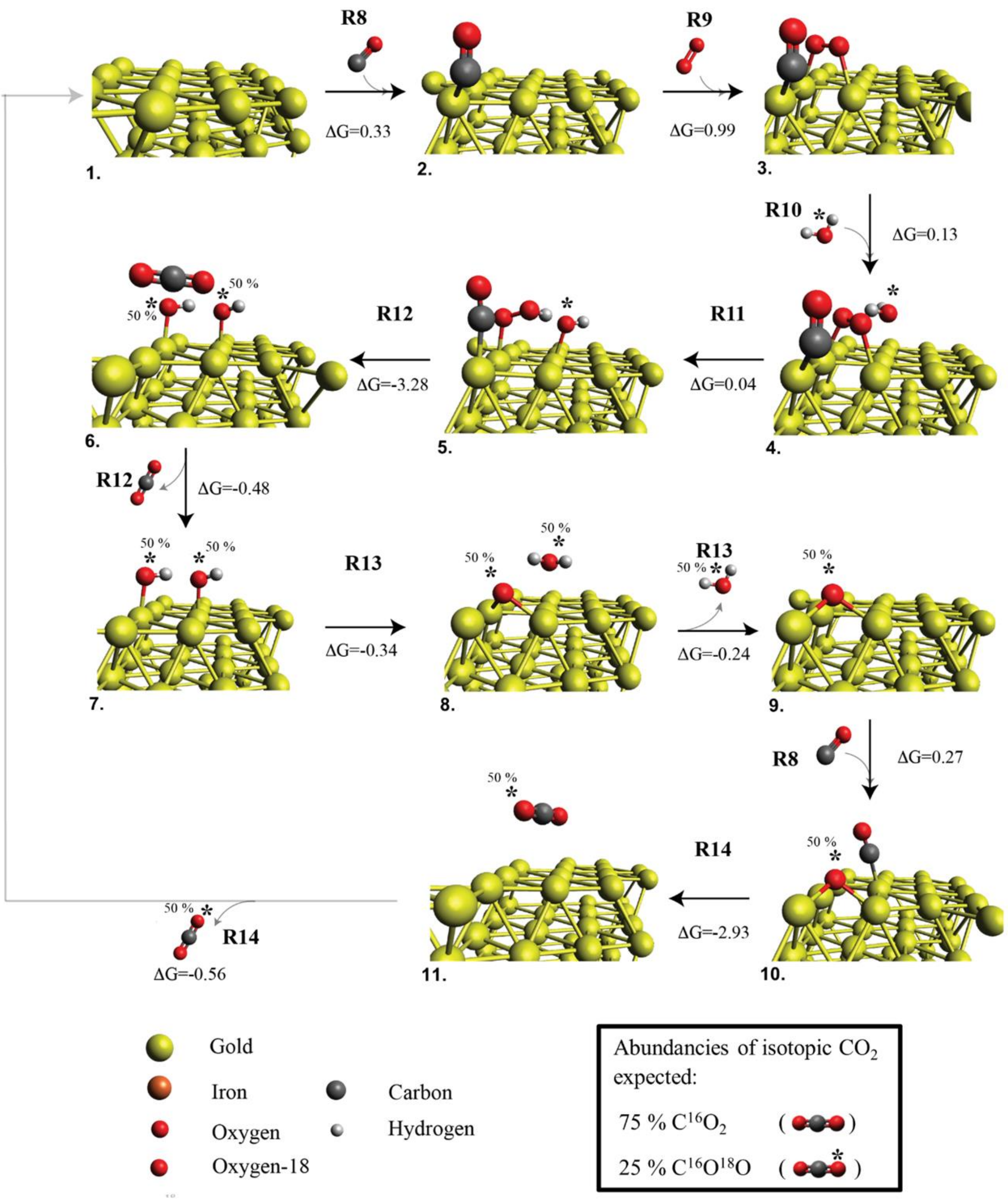

Figure S15. Reproduction of Figure S4, but with $H_2{}^{18}O$ instead of $H_2{}^{16}O$. The figure illustrates how the abundancies of different isotopic CO_2 species can be predicted from Iglesia's proposed reaction mechanism[2] (Scheme 2 main paper, and Scheme S2) of water-promoted CO oxidation with $C^{16}O$, $^{16}O_2$ and $H_2{}^{18}O$. Here with the mechanism running on Au(111). It can thus be predicted the mechanism would result in 75 % $C^{16}O_2$ and 25 % $C^{16}O^{18}O$ (as is the case if the mechanism is allowed to run on Au-γ-Fe_2O_3, see Figure S14). Reaction free energies and activation free energies in eV. Structures labelled **1. – 11.**

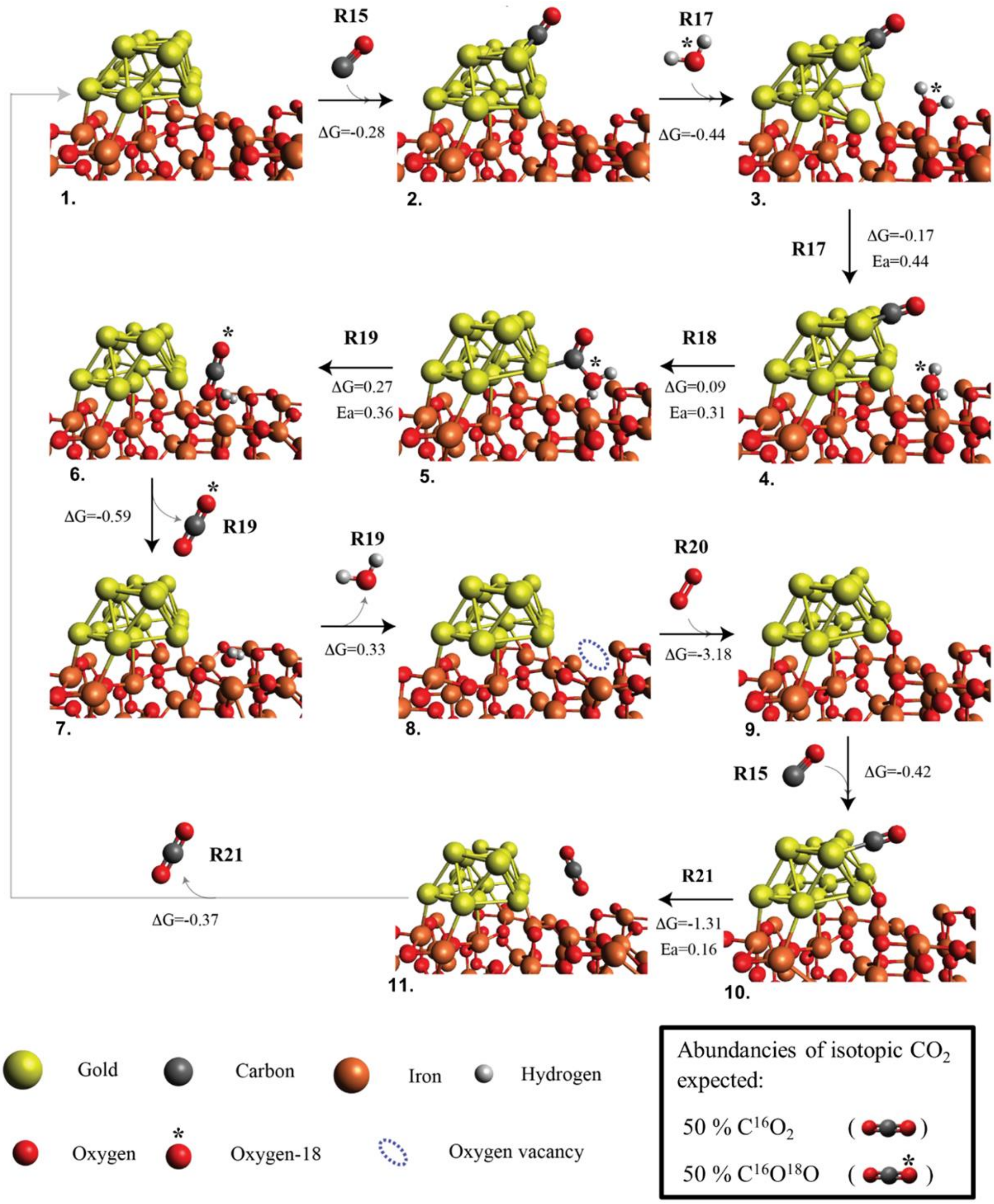

Figure S16. Reproduction of Figure S5, but with $H_2{}^{18}O$ instead of $H_2{}^{16}O$. The figure illustrates how the abundancies of different isotopic CO_2 species can be predicted from our proposed w-MvK reaction mechanism[2] (Scheme 3 and Figure 3 main paper, and Scheme S3) of water-promoted CO oxidation with $C^{16}O$, $^{16}O_2$ and $H_2{}^{18}O$. It can thus be predicted the w-MvK mechanism would result in 50 % $C^{16}O_2$ and 50 % $C^{16}O^{18}O$. Reaction free energies and activation free energies in eV. Structures labelled **1. – 11.**

References

(1) Zanella, R.; Giorgio, S.; Shin, C. H.; Henry, C. R.; Louis, C. Characterization and Reactivity in CO Oxidation of Gold Nanoparticles Supported on TiO2 Prepared by Deposition-Precipitation with NaOH and Urea. *J Catal* **2004**, *222* (2), 357–367. https://doi.org/10.1016/j.jcat.2003.11.005.

(2) Ojeda, M.; Zhan, B. Z.; Iglesia, E. Mechanistic Interpretation of CO Oxidation Turnover Rates on Supported Au Clusters. *J Catal* **2012**, *285* (1), 92–102. https://doi.org/10.1016/j.jcat.2011.09.015.

(3) Schubert, M. M.; Hackenberg, S.; van Veen, A. C.; Muhler, M.; Plzak, V.; Behm, R. J. CO Oxidation over Supported Gold Catalysts -"Inert" and “Active” Support Materials and Their Role for the Oxygen Supply during Reaction. *J Catal* **2001**, *197* (1), 113–122. https://doi.org/10.1006/jcat.2000.3069.

(4) Kresse, G.; Furthmüller, J. Efficient Iterative Schemes for Ab Initio Total-Energy Calculations Using a Plane-Wave Basis Set. *Phys Rev B* **1996**, *54* (16), 11169–11186. https://doi.org/10.1103/PhysRevB.54.11169.

(5) Perdew, J. P.; Burke, K.; Ernzerhof, M. Generalized Gradient Approximation Made Simple. *Phys Rev Lett* **1996**, *77* (18), 3865–3868. https://doi.org/10.1103/PhysRevLett.77.3865.

(6) Blochl, P. E. Projector Augmented-Wave Method. *Phys Rev B* **1994**, *50* (24), 17953–17979. https://doi.org/https://doi.org/10.1103/PhysRevB.50.17953.

(7) Blochl, P. E.; Jepsen, O.; Andersen, O. K. Improved Tetrahedron Method for Brillouin-Zone Integrations. *Phys Rev B* **1994**, *49* (23), 16223–16233. https://doi.org/https://doi.org/10.1103/PhysRevB.49.16223.

(8) Methfessel, M.; Paxton, A. T. High-Precision Sampling for Brillouin-Zone Integration in Metals. *Phys Rev B* **1989**, *40* (6), 3616–3621.

(9) Hjorth Larsen, A.; JØrgen Mortensen, J.; Blomqvist, J.; Castelli, I. E.; Christensen, R.; Dułak, M.; Friis, J.; Groves, M. N.; Hammer, B.; Hargus, C.; Hermes, E. D.; Jennings, P. C.; Bjerre Jensen, P.; Kermode, J.; Kitchin, J. R.; Leonhard Kolsbjerg, E.; Kubal, J.; Kaasbjerg, K.; Lysgaard, S.; Bergmann Maronsson, J.; Maxson, T.; Olsen, T.; Pastewka, L.; Peterson, A.; Rostgaard, C.; SchiØtz, J.; Schütt, O.; Strange, M.; Thygesen, K. S.; Vegge, T.; Vilhelmsen, L.; Walter, M.; Zeng, Z.; Jacobsen, K. W. The Atomic Simulation Environment - A Python Library for Working with Atoms. *Journal of Physics Condensed Matter* **2017**, *29* (27). https://doi.org/10.1088/1361-648X/aa680e.

(10) Bengtsson, L. Dipole Correction for Surface Supercell Calculations. *Phys Rev B* **1999**, *59* (19), 12301–12304. https://doi.org/https://doi.org/10.1103/PhysRevB.59.12301.

(11) Henkelman, G.; Uberuaga, B. P.; Jónsson, H. Climbing Image Nudged Elastic Band Method for Finding Saddle Points and Minimum Energy Paths. *Journal of Chemical Physics* **2000**, *113* (22), 9901–9904. https://doi.org/10.1063/1.1329672.

(12) Pecharromfin, C.; Gonzfilez-Carrefio, T.; Iglesias, J. E. The Infrared Dielectric Properties of Maghemite, Gamma-Fe2O3, from Reflectance Measurement on Pressed Powders. *Phys Chem Minerals* **1995**, *22*, 21–29. https://doi.org/https://doi.org/10.1007/BF00202677.

(13) Dos Santos, E. C.; Lourenço, M. P.; Pettersson, L. G. M.; Duarte, H. A. Stability, Structure, and Electronic Properties of the Pyrite/Arsenopyrite Solid-Solid Interface-A DFT Study. *Journal of Physical Chemistry C* **2017**, *121* (14), 8042–8051. https://doi.org/10.1021/acs.jpcc.7b02642.

(14) Halldin Stenlid, J.; Campos dos Santos, E.; Johansson, A. J.; Pettersson, L. G. M. Properties of Interfaces between Copper and Copper Sulphide/Oxide Films. *Corros Sci* **2021**, *183*. https://doi.org/10.1016/j.corsci.2021.109313.

(15) Yu, X.; dos Santos, E. C.; White, J.; Salazar-Alvarez, G.; Pettersson, L. G. M.; Cornell, A.; Johnsson, M. Electrocatalytic Glycerol Oxidation with Concurrent Hydrogen Evolution Utilizing an Efficient MoOx/Pt Catalyst. *Small* **2021**, *17* (44). https://doi.org/10.1002/smll.202104288.

(16) NIST-JANAF Thermochemical Tables. **2013**. https://doi.org/10.18434/T42S31.

(17) Wang, Y. G.; Cantu, D. C.; Lee, M. S.; Li, J.; Glezakou, V. A.; Rousseau, R. CO Oxidation on Au/TiO2: Condition-Dependent Active Sites and Mechanistic Pathways. *J Am Chem Soc* **2016**, *138* (33), 10467–10476. https://doi.org/10.1021/jacs.6b04187.

(18) Aylward, G.; Findlay, T. *SI Chemical Data*, 5th ed.; Wiley: Milton, 2002; Vol. 5.

(19) Saavedra, J.; Pursell, C. J.; Chandler, B. D. CO Oxidation Kinetics over Au/TiO2 and Au/Al2O3 Catalysts: Evidence for a Common Water-Assisted Mechanism. *J Am Chem Soc* **2018**, *140* (10), 3712–3723. https://doi.org/10.1021/jacs.7b12758.

(20) Jin, C.; Zhou, Y.; Han, S.; Shen, W. Water-Assisted Low-Temperature Oxidation of CO at the Au-Fe2O3Interface. *Journal of Physical Chemistry C* **2021**, *125* (47), 26031–26038. https://doi.org/10.1021/acs.jpcc.1c07995.
